# The development and utility of frameworks designed to evaluate research capacity building initiatives in healthcare settings: a methodological review

**DOI:** 10.1186/s12961-026-01511-3

**Published:** 2026-07-16

**Authors:** Olivia A. King, Ella Ottrey, Michele Conlin, Sze Lin Yoong, Anna Wong Shee, Emma West, Laura Alston, Joanne Porter, Catherine E. Huggins

**Affiliations:** 1 Western Alliance Academic Health Science Centre, Warrnambool, Australia; 2 Barwon Health, Geelong, Australia; 3 East Grampians Health Service, Ararat, Australia; 4 Institute for Health Transformation, Deakin University, Geelong, Australia; 5 Grampians Health, Ballarat, Australia; 6 Deakin Rural Health, Warrnambool, Australia; 7 Deakin University, IMPACT, Geelong, Australia; 8 Colac Area Health, Colac, Australia; 9 Collaborative Evaluation and Research Centre, Federation University, Churchill, Australia

**Keywords:** Research capacity building, Evaluation, Methodological review, Health services research

## Abstract

**Background:**

Building research capacity in health settings is essential for improving the relevance and application of health research. Rigorous evaluation of research capacity in health settings optimizes the development, implementation and improvement of such initiatives. Cooke’s 2005 framework was the earliest peer-reviewed framework to evaluate research capacity in health settings. While subsequent frameworks exist, it is not clear how they were developed and are used in practice. This review investigates the development, composition and utilization of peer-reviewed frameworks designed to evaluate research capacity in health settings.

**Methods:**

This two-phased methodological review was informed by the JBI scoping review methodology. Phase 1 involved a systematic search of seven databases on 1 May 2025 to identify peer-reviewed frameworks for evaluating research capacity. Phase 2 involved a forward citation search of included frameworks using Google Scholar, finalized on 18 September 2025, to identify the number and nature of citing studies. Data extraction and narrative synthesis were informed by the review aims.

**Results:**

Phase 1 database searches yielded 2581 unique citations, of which 8 met the inclusion criteria. An additional framework was identified through citation searching, totalling 9 frameworks. The frameworks were developed via retrospective processes (reviews of literature and local data) or prospective methods (engagement workshops). Several were informed by earlier frameworks. Frameworks typically comprised an overarching structure, substructural components and a series of outcome indicators. Framework authors defined research capacity building in nuanced ways.

Phase 2 forward citation searching of the nine frameworks revealed 881 citations. Of these, 37 citing articles met the inclusion criteria. Most (31/37) citing studies utilized Cooke’s 2005 framework. Frameworks were largely used to inform data collection and analysis, and most citing authors only used only some framework components. Limitations and strengths of the frameworks related to the development process and practical application were identified by framework and citing authors.

**Conclusions:**

This review facilitated new insights into the development, characteristics and utilization of frameworks to evaluate research capacity building in health settings. There are nine peer-reviewed, published frameworks, several informed by and expanding on earlier frameworks, which is essential for advancing the evaluation of research capacity in health settings.

**Supplementary Information:**

The online version contains Supplementary Material available at 10.1186/s12961-026-01511-3.

## Background

Building the capacity of individual health professionals and health organizations to inform, conduct and use research is critical to addressing problems in health practice and increasing the delivery of evidence-based and higher-quality care [1–3]. Increased engagement with research enhances quality of care and reduces the gap between evidence and practice [1, 2]. To achieve this aim, concerted efforts to build research capacity in health settings are undertaken in high-, middle- and lower-income countries [3–5]. Different research capacity building (RCB; also referred to as research capacity strengthening and research capacity development) [4, 6] strategies are described in the literature. These strategies include research training workshops, webinars, research mentoring, fellowships, seed and larger grant funding for clinician-led research, clinical–academic partnerships, health organization-embedded research roles and organizational research strategies [7, 8]. RCB programmes and initiatives often utilize multiple strategies in synergy to affect increased individual and organizational research capacity [2, 7].

Decisions about the effectiveness of RCB programmes, concomitant funding and resource allocation and programme development in health settings rely on robust evaluation [9]. Evaluation is essential in the context of health interventions, health promotion programmes [9, 10] and health professions education [11], to determine whether these programmes are effective in achieving the improvements in capacity and outcomes, as well as whether and what modifications need to be made. Evaluation frameworks provide for a structured and systematic approach to evaluating the implementation and outcomes of a programme or initiative, and progress towards realizing a defined goal [9, 12].

The multifaceted nature of RCB programmes and initiatives makes evaluating the outcomes and impacts difficult; particularly when it comes to identifying mechanisms or causal links between programme elements and outcomes [2, 13]. Furthermore, health settings are complex, dynamic environments wherein there are many uncontrollable and interrelated factors that influence the outcomes achievable within a setting at a given time [14]. Indeed, previous reviews of the RCB literature have highlighted the inconsistencies and complexities inherent in evaluating RCB programmes delivered in health settings [2, 7, 15]. Other factors that are known to hinder RCB programme evaluation include differences in the anticipated outcomes and priorities held by various interest-holders [16]. These differences in perspectives mean efforts to demonstrate impact in RCB rarely satisfy all interest-holders and point to the need for a robust framework to guide evaluation.

Bilardi et al.’s [3] systematic review of tools to measure individual-level research capacity in healthcare workers highlighted the predominant use of a range of questionnaire-based tools, with little evidence of the tools evaluating capacity development over time [3]. The authors elucidated the potential for standardized evaluation tools to afford a level of consistency and opportunities for direct comparison of outcomes across programmes and initiatives [3]. Evaluation frameworks, which are distinct from evaluation tools, serve a slightly different, yet equally important role in guiding and informing robust evaluation studies and facilitating the investigation of mechanisms for change. That is, eliciting detailed understandings of how programmes worked or did not work [12] and therefore what improvements are needed.

In 2005, Cooke [17] published a seminal piece in the emerging RCB literature, in her pursuit to “establish a framework for planning and measuring progress, and to initiate a debate about identifying what are appropriate outcomes for RCB, not simply to rely on things that are easy to measure” [17; p. 2]. Since then, several RCB evaluation frameworks have been published within and for different geographical, academic and healthcare contexts. Recent reviews of research education and training programmes, and research engagement by healthcare providers and organizations, indicate that evaluation rarely considers practice-based, organization-level and longer-term outcomes and impacts, and that the evidence on research engagement is primarily opinion-based and descriptive in nature [7, 18]. Some reasons suggested for these shortcomings in evaluating RCB programmes are that not all evaluations are informed by a framework, that practice-based and organization-level impacts take a long time to achieve and that resource allocation may be insufficient to support longer-term evaluation [7].

Evaluating research capacity and RCB programmes is critical for optimizing, demonstrating the value of and sustaining programmes delivered in often resource-constrained healthcare settings [4, 19]. Addressing the question of how have existing frameworks for evaluating RCB been developed and applied in practice may provide an opportunity to better understand the real-world impact of such programmes. This review therefore analyses the development, components and subsequent utilization of peer-reviewed published frameworks designed to evaluate research capacity in health settings, with a view to develop insights into how to utilize these frameworks and balance high-quality robust evaluation with context and resource constraints.

## Review aims

This methodological review aims to identify: (1) the processes utilized in the development of frameworks for evaluating RCB programmes and initiatives implemented in health settings; (2) the key components of these frameworks; (3) the underpinning evidence and theories; and (4) utility of the frameworks as evidenced by forward citations and the nature of their use by other researchers. A methodological review was selected over other common review methodologies (e.g. systematic or scoping review) as it is best aligned with the aims of the study. This approach enables critical review of frameworks with respect to how their development considered interest-holders, context and how they were applied in evaluation practice. This review will synthesize the strengths and limitations of the published frameworks and their use in evaluation practice. In addition, it will produce recommendations on the further development and use of RCB evaluation frameworks.

## Methods

This methodological review draws on the principles of scoping reviews [20, 21] and is reported in line with the Preferred Reporting Items for Systematic Reviews and Meta-Analyses extension for Scoping Reviews (PRISMA-ScR) guidelines (Additional File 1 PRISMA-ScR Checklist [22]). JBI scoping review methodology informed the review protocol [23]. The review questions, inclusion and exclusion criteria, and search strategies were developed prior to running the search (Additional File 2 Scoping Review Protocol). This methodological review comprised two phases: phase 1, a systematic search of the literature to identify peer-reviewed published frameworks designed to evaluate research capacity in health settings, and phase 2, forward citation searches of the frameworks identified in phase 1 to ascertain the frequency and nature of their use in the peer-reviewed published literature.

### Search strategy

#### Phase 1

The initial search was designed to systematically identify peer-reviewed, published frameworks to evaluate research capacity in health settings. The JBI three-step search strategy was applied. The authors identified a set of key articles on the basis of their knowledge of peer-reviewed, published frameworks to evaluate research capacity. These articles were used to identify key search terms. In consultation with the research librarian (C.F.; see Acknowledgements), preliminary scoping searches were conducted to test the search terms and strategy and refine the final search terms. A tailored search strategy was developed for each academic database (Additional File 3 Search Strategy).

Seven academic databases were searched: Ovid MEDLINE, CINAHL, PsycInfo, ERIC, Embase, Scopus and Web of Science. No date limits were applied, and all literature published until the final database search was conducted (1 May 2025) was included. Final database searches were conducted by one author in consultation with the research librarian. Searches of the reference lists of included articles and forward citation searches using Google Scholar were undertaken. This led to the identification of one further research capacity evaluation framework.

Grey literature and reports describing research capacity evaluation frameworks were excluded, as phase 2 of this methodological review was a forward citation search in the peer review literature. To our knowledge, there are no existing search engines that can systematically track the citations of grey literature.

#### Phase 2

A series of forward citation searches for each of the framework articles (identified in phase 1) was conducted via Google Scholar. These citation searches were initially conducted on 26 May 2025 and were repeated on 18 September 2025 to ensure recent citing articles were included in the review.

### Eligibility criteria

#### Phase 1

The inclusion and exclusion criteria for articles retrieved through the academic database searches were developed and presented in line with the recommendations of Munn et al. [24] and as described in Clarke et al. [25]. Criteria are presented in Table 1.

**Table 1:** Inclusion and exclusion criteria for phase 1 database searches

Criteria	Inclusion	Exclusion
Types of studies	All types of studies that describe the development of new or modified evaluation frameworks, models or strategies to guide the evaluation of outcomes or impacts of research capacity building programmes, strategies or initiatives implemented in health settings*	Individual programme evaluations that do not introduce or describe the development of a new evaluation framework, construct or organizing theory or structure; generic evaluation frameworks PhD theses Systematic reviews
Types of data	All forms of data used or described as part of the development of a framework to evaluate research capacity building programmes delivered in healthcare settings (qualitative, quantitative, mixed, newly generated/primary data and secondary data); objective (e.g. metrics) and subjective (e.g. views/experiences) data	Papers presenting evaluation data with no reference to the development of the evaluation framework
Types of methods	Qualitative methods (interviews, focus groups, open text surveys, narrative or integrative literature reviews) Quantitative methods (surveys, scales, meta-syntheses) Consensus methods (Delphi studies, roundtable discussions, workshops) Mixed-methods approaches to developing frameworks	None
Types of outcome measures	Papers that describe any outcome measures as part of a research capacity building programme evaluation framework	None

*Health settings are defined as settings in which healthcare professionals work and deliver care and provide services to consumers/patients (e.g. hospitals, community health services, family practice, and clinics or surgeries)

#### Phase 2

Articles were included if they reported on an evaluation of research capacity in health professionals or health settings, were peer-reviewed and used the cited framework(s) to inform the evaluation methods or presentation of the results (i.e. the framework was cited in the Methods and/or Results sections). Articles were excluded if the framework was cited for the purpose of framing the study and/or comparing and contrasting findings (i.e. the framework was cited in the introduction or discussion sections only), if they did not report on an evaluation of research capacity, if the study evaluated research capacity in academic or other non-health settings and if the article was not peer-reviewed.

### Study selection, quality appraisal and data extraction

#### Phase 1

Articles retrieved from the database searches were imported into Covidence (Veritas Health Innovation, Melbourne, Australia) for duplicate removal and review. Titles and abstracts were reviewed independently by two authors, and conflicts were resolved by a third author. Similarly, full texts were reviewed by two authors, and the reasons for exclusion were documented (Additional File 4 Excluded Framework Articles). This initial search and reviewing led to the identification of a set of peer-reviewed, published articles describing frameworks to evaluate research capacity in health settings.

#### Phase 2

Phase 2 involved a cursory analysis of how the frameworks have been used in citing articles. A bespoke tool comprising structured, objective criteria (as described above) was developed and applied to citing articles. Two authors (the lead and a second author) reviewed 10% of the articles and achieved 100% agreement. The lead author reviewed the remainder of the citing articles using the tool objectively, consulting with the research team on encountering any ambiguous studies, ensuring both rigour and timeliness of this phase. Data extraction tables were developed for the two phases of the review in accordance with the review aims. Data were extracted from each of the included framework articles by one author and reviewed by a second author for consistency and accuracy. Data from each of the citing articles were extracted by one author, with data independently extracted from 66% of articles by another member of the research team. Formal quality appraisal was not undertaken as part of this review, in line with scoping review methodology [24]. Data extracted and tabulated from the evaluation framework articles and citing articles were synthesized using a descriptive approach guided by the review aims. The outcome indicators, which where present in the frameworks, were categorized using a content analysis approach [26].

## Results

### Phase 1

**Fig. 1:**
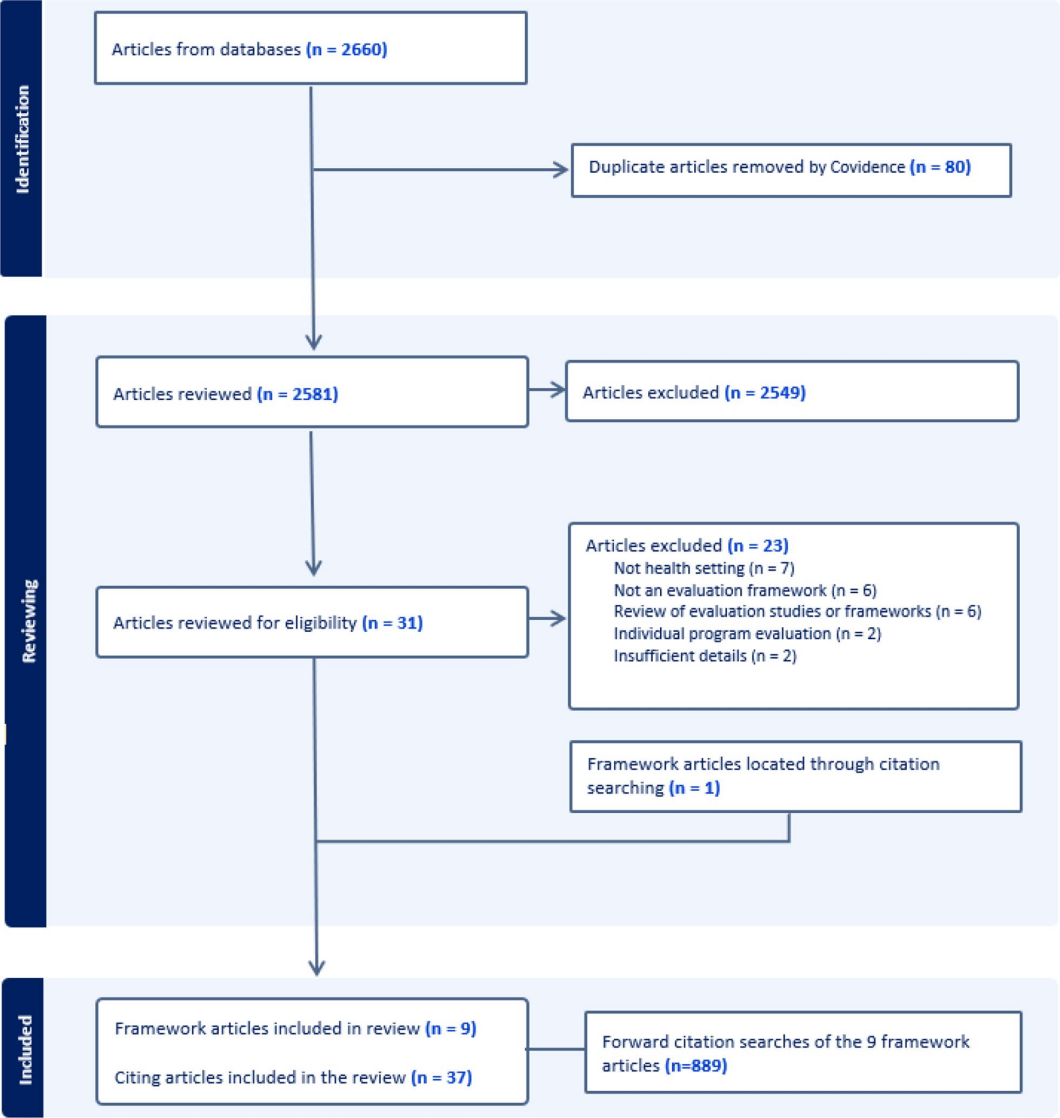
PRISMA flow diagram

Of the 2581 unique articles retrieved via database searches, 32 full texts were reviewed for potential inclusion in the review. Of these, eight articles met the inclusion criteria, with a summary of the reasons for exclusion shown in Fig. 1. Through hand-searching reference lists of the eight articles [27], an additional framework article was identified. In total, nine peer-reviewed published frameworks were identified. Seven were published in open-access journals [6, 17, 28–32], and two were accessible via institutional or personal subscription [33, 34].

### Phase 2

Forward citation searches of the nine frameworks led to the identification of 881 citing studies. Of these, 37 met the inclusion criteria.

### Defining research capacity building

Seven of the nine framework articles included an explicit definition of research capacity building or relevant synonym (e.g. research capacity development or strengthening). Two frameworks [30, 31] were informed by a definition adopted from Golenko et al. [35], which refers to individual and organizational ability to undertake useful research. Sarre’s framework [33] adopted an earlier definition published by Trostle [36] in 1992, which is similar to Golenko’s [35] definition. In the earlier framework article, Cooke [17] cited two published definitions before putting forth their own, which emphasizes that capacity building is not the end itself, but rather a means to producing impactful research. Cooke’s later framework [6] uses a similar definition. While two authors [29, 32] did not explicitly state the definition their frameworks aligned with, they did describe the overarching purpose and longer-term aims of RCB (i.e. to improve health outcomes). Six definitions or descriptions of RCB referred to the relevance of organizational or institutional capacity [17, 29–31, 33, 34], and all nine referred to promoting the conduct of health research that is used or useful.

### Development of research capacity evaluation frameworks

Frameworks were published between 2005 and 2023. Their development and components are summarized in Table 2. Seven frameworks were developed using one or more of the following retrospective methods: review and analysis of the research capacity building literature [17, 28, 31, 32]; review and analysis of case studies/best-practice examples [28–30, 32]; authors’ experience and reflections [6, 17, 32]; and review of local evidence [32]. Three were developed using prospective methods, such as interest-holder engagement [28, 33, 34]. One framework was developed using both retrospective and prospective methods [28]. Six frameworks were informed by earlier published frameworks [6, 28–30, 32, 33]. Four frameworks were developed for or within low- and middle-income country contexts [28–31]. One framework was developed in a regional setting in a high-income country [34].

**Table 2:** Framework development

Lead author, year of publication Name of framework	Country(ies) developed in	Geographic context of development/application	Definition of research capacity building	Mechanism(s) of development	Underpinning theory or framework	Strengths and limitations of framework as reported by author
Cooke, 2005 [17] Framework to evaluate research capacity building in health care	•England	•High-income country •Metropolitan location	“RCB can therefore be seen as a means to an end, the end being ‘useful’ research that informs practice and leads to health gain, or an end in itself, emphasising developments in skills and structures enabling research to take place” [17; p. 44]	•Analysis of the literature, including policy documents and research studies •The experience of one Research Development Support Unit in the United Kingdom	•Two dimensions: 1. Four structural levels of development activity 2. Six principles of capacity building	Strengths •Combined use of process and outcome indicators can help unpack the mechanisms of RCB Limitations •Framework not yet tested •Author expects additional criteria will be developed, and used •Considerations for the use of the framework include time constraints, resources and the purpose of each evaluation
Bates, 2006 [28] Komfo Anokye Teaching Hospital (KATH) tool	•Ghana	•Lower-middle-income country	“For health research, the goal of building capacity is thus to improve the ability to conduct research, to use results effectively, and to promote demand for research” [28; p. 1224]	•Review of literature related to existing tools and models •Analysis of published best-practice examples •Workshop with hospital stakeholders (health professionals, chief executive, medical director and heads of department) to adapt the framework into a functional evaluation tool	•Previously published framework: Silimperi et al. [37] Framework for institutionalizing quality assurance	Strengths •Enables institutions in developing countries to set its own RCB priorities, control its activities and evaluate progress accordingly Limitations •Components are missing from the framework that relate to relationships (e.g. dialogue between scientists and non-scientists, health workers) •Tool focused solely on organizational capacity
Sarre, 2009 [33] Indicators for measuring research capacity development in primary care organizations	•England	•High-income country •Metropolitan location	“RCD [research capacity development] is ‘a process of individual and institutional development which leads to higher levels of skills and greater ability to perform useful research’” cites Trostle [36] [33; p. 244]	•Stakeholder engagement over two stages: 1. Five workshops with > 70 participants representing a range of people with a research interest 2. Consensus-building activity following a modified nominal group technique via questionnaire and meeting	•Cooke’s [17] framework to evaluate research capacity building in health care	Strengths •Builds on Cooke’s [17] framework contributing three additional domains, with indicators that are supported by the existing RCB literature Limitations •Inclusion of only seven experts in consensus building limits the findings to their views and experiences •Study and participants set in the North of England where the context may not reflect that of other settings •Feasibility of indicators not investigated
Bates, 2011 [29] Indicators of sustainable capacity building for health research	•Ghana •Drawing on data from programmes and stakeholders based in multiple low- and middle-income countries (LMICs)	•Low- and middle-income countries	No explicit definition of RCB; however, authors state, “Strengthening the capacity of health systems is closely linked to building research capacity because high quality research is essential to identify and prioritize health needs, and to develop appropriate strategies to improve health outcomes” [29; p. 2]	•Analysis of four case studies; each case set in a different African country, focused on a different health topic, operating at a different level of the health service •Previously published framework [28] was used to analyse the four cases	•Previously published framework: Bates KATH tool [28]	Strengths •Transferable and generic indicators that could be used with project-specific indicators for a flexible approach to evaluation Limitations •Authors’ close involvement with the cases may have introduced some biases
Cole, 2014 [30] Indicators for tracking programmes to strengthen health research capacity in lower- and middle-income countries	•Canada, United Kingdom and Switzerland-based researchers •Drawing on data from programmes and stakeholders based in multiple low- and middle-income countries (LMICs)	•Low- and middle-income countries	“RCS [research capacity strengthening] has been defined as a ‘process of individual and institutional development which leads to higher levels of skills and greater ability to perform useful research’” cites Golenko et al. [35] [30; p. 1]	•Case study and analysis (review of 18 reports from 12 evaluations of funded RCB programmes delivered in LMICs)	•Previously published framework: ESSENCE Planning, Monitoring and Evaluation framework	Strengths •Indicators can be tailored to the aims of different initiatives and contexts Limitations •Not all programme funders provided reports •Labour-intensive nature of data extraction and analysis limited the depth of analysis of the reports included
Murphy, 2015 [34] Management strategy analysis	•Australia	•High-income country •Regional location	“Research capacity building (RCB) refers to individual and organizational developments which lead to greater ability to access, conduct, and apply useful research” [34; p. 14]	•Stakeholder engagement via a survey of health service staff using a pre-existing tool and a workshop/ranking exercise with senior health service leaders	•Existing RCB literature supporting three essential conditions for sustainable RCB: 1. Collaborative/partnership approach 2. Involvement of senior health service leadership 3. Engagement of research-interested staff	Strengths •Ensures the alignment between the health service’s organizational strategic plan and the RCB strategy Limitations •Nil reported
Pulford, 2020 [31] Indicators for measuring the outcome and impact of research capacity strengthening initiatives	•England •Data from programmes delivered in LMICs	•Low- and middle-income countries	“Research capacity strengthening (RCS) has been defined as the ‘process of individual and institutional development which leads to higher levels of skills and greater ability to perform useful research’” cites Golenko et al. [35] [31; p. 1]	•Structured review of RCB indicators described in the published and grey literature	•Output indicators, outcome indicators and impact indicators informed via a structured review and synthesis of existing literature and reports	Strengths •Comprehensive list of common indicators of research capacity used in practice Limitations •Lack of universal definition of RCB meant the search and retrieval of all relevant literature was limited •RCB indicators described in languages other than English were excluded •Quality measure did not account for relevance or feasibility and was biased towards quantitative indicators
Cooke, 2021 [6] Research Capacity Development for impact (RCDi) framework	•England	•High-income country •Metropolitan location	“Capacity building is seen not an end in itself, but a means to an end: that is it is designed to develop and produce research that is used and impactful” [6; p. 1]	•Based on Cooke’s [17] framework, which has been further developed iteratively and adapted over time	•Cooke’s [17] framework to evaluate research capacity building in health care	Strengths •Guides research–practice partnerships across four structural levels, and through principles embedded in the capacity building interventions Limitations •Nil reported
Sabey, 2023 [32] Framework to guide the evaluation of training in research skills for health and care professionals	•England	•High-income country •Metropolitan location	No explicit definition of RCB; however, authors state, “The drive to increase research capacity in health-care reflects the belief that developing the skills of health-care professionals to use and undertake research improves health outcomes and advances healthcare” [32; p. 84]	•Review and analysis of another Applied Research Collaboration’s unpublished model of RCB impact assessment •Authors’ experiences of RCB to further define areas of impact •Literature review and analysis •Further documentary or report analysis (participant course evaluation reports)	•Kirkpatrick model •Cooke’s [17] framework to evaluate research capacity building in health care	Strengths •Structure to help develop programmes with a focus on measuring medium- and longer-term outcomes •Comprises many transferable elements that could be adapted and used in other contexts Limitations •Nil reported

### Key characteristics and components of research capacity evaluation frameworks

Eight of the frameworks comprise overarching structural components [6, 17, 28–32, 34], all nine frameworks have substructural components, and eight include a series of outcome measures, indicators or example criteria ([17, 28–34]; see Table 3 and Additional File 5 List of all example indicators and outcomes for a comprehensive summary of the latter).

**Table 3:** Framework structures, components and citation frequencies

Lead author, year of publication Name of framework	Overarching structural components	Substructural components	Outcome measures/indicators (see Additional File 5 List of all example indicators and outcomes)	Forward citations (*n*)	Citing studies that met the inclusion criteria* (*n*)**
Cooke, 2005 [17] Framework to evaluate research capacity building in health care	Four structural levels of development activity: •Individual •Teams •Organizational •Supra-organizational	Six principles of capacity building: •Building skills and confidence •Close to practice •Linkages, collaborations and partnerships •Appropriate dissemination and impact •Continuity and sustainability •Infrastructure	92 examples of suggested criteria across the six principles and four structural levels of development	481	31
Bates, 2006 [28] KATH tool	Four phases of RCB progress: •Awareness •Implementation •Expansion •Consolidation	Activities Awareness: •Baseline assessment of quality of care •Raise awareness of the need for improved institutional research culture Implementation: •Establish research training and develop into accredited diploma Expansion: •Enrolment of clinical and nonclinical participants •National organizations involved in programme •National dissemination meeting Consolidation: •Research activity integrated into daily practice in organization •Research support team in place	Five indicators of progress across the four phases and 11 activities	144	1
Sarre, 2009 [33] Indicators for measuring research capacity development in primary care organizations	N/A	Nine principles of RCB: •Infrastructure •Linkages and partnerships •Skills development •Dissemination •Research activity •Close to practice •Continuity and sustainability •Leadership •Research culture	26 indicators across the nine principles	56	5
Bates, 2011 [29] Indicators of sustainable capacity building for health research	Four phases of RCB progress: •Awareness •Experiential •Expansion •Consolidation	Activities Awareness: •Recognition of lack of research capacity •Stakeholders agree to support research capacity building activities •Identification of need for research to be implemented Experiential: •RCB activities focused on individuals •Exploration of formal and informal routes for using research findings in practice/policy •Formal plans for addressing capacity needs •Preliminary models for RCB are tested and adapted •Strategies for ensuring relevant policies in place or updated Expansion: •Efforts to influence practice and policy •Focus broadens to institutions and systems •Capacity building integrated into existing structures •Sustainable funding pursued •Peer-reviewed and published research Consolidation: •Expansion beyond initial project and institution •Southern partners lead funding bids •Southern partners manage project and budget	13 generic indicators derived from activities	101	2
Cole, 2014 [30] Indicators for tracking programmes to strengthen health research capacity in lower- and middle-income countries	Three structural levels of outputs and outcomes: •Individual-level indicators of outputs and outcomes •Institutional-level indicators of outputs and outcomes •National–international level indicators of outputs and outcomes	Pathways to change Individual level: •Research skills training activities •Mentoring activities •Scientific conference and workshop activities •Course and curricula development activities Institutional level: •Human resources strengthening activities •Activities for strengthening research infrastructure and management •Scientific collaboration activities National–international level: •Engagement and communication activities for research uptake •Activities to develop national health research systems or scientific councils •Networking activities for researchers and/or research users	95 activities, outputs and outcomes across the three structural levels	45	0
Murphy, 2015 [34] Management strategy analysis	Timeframe: •Immediate •Intermediate •Ultimate	Objectives •Research-supportive organizational policies and procedures •Staff knowledge, skills and confidence to access and use research •Involvement of health service staff in research activity •Research communication within health service •Research informs organizational governance documents •Health service staff work satisfaction •Use of research in health practice	14 success indicators	6	0
Pulford, 2020 [31] Indicators for measuring the outcome and impact of research capacity strengthening initiatives	Three structural levels: •Individual-level categories and indicators •Institutional-level categories and indicators •Systemic level categories and indicators	Outcome indicator categories •Bibliometrics •Collaboration activities •Knowledge translation •Recognition •Research funding •Research management systems •Skills/knowledge •Other	70 example indicators across the eight outcome indicator categories and three structural levels	24	0
Cooke, 2021 [6] Research Capacity Development for impact (RCDi) framework	Three structural levels of development activity: •Individual •Organizational •Health and social care systems	Seven principles of capacity building: •Skills and confidence building •Co-production •Linkages and collaborations •Actionable dissemination •Sustainability and leadership •Infrastructure •Ownership and responsibilities	N/A	32	4
Sabey, 2023 [32] Framework to guide the evaluation of training in research skills for health and care professionals	Levels of impact: •Individual level •Group or organization level •Health and care system level Timeframe: •Immediately post-training •Medium term (3–6 months •Longer term (> 12 months)	Impact domains Individual level: •Knowledge and understanding •Confidence •Capability •Change in work practices/behaviours •Career development Group or organization level: •Workforce research/evaluation capability •Cascade/sharing •Social capital •Change in organizational processes •Organizational research culture Health and care system level: •Cross-system research/evaluation/policy collaboration •Sustainability	12 types of impact mapped to the impact domains aligned with the three impact levels and three timeframe categories	0	0

*Inclusion criteria: articles that reported on an evaluation of research capacity in health professionals or health settings, were peer-reviewed and used the cited framework(s) to inform the evaluation in some way

**Some articles cited more than one framework

Of the eight frameworks with overarching structural components, five of these were informed by Bronfenbrenner’s [37] ecological theory (i.e. individual, team, organizational/institutional, supra-organizational/national/system structural levels; [6, 17, 30–32]). Two frameworks [28, 29] were informed by organization theory and phases of developmental progress [38–40]: awareness, implementation (or experiential), expansion, and consolidation. Two frameworks were informed by timeframe (i.e. short, medium and longer term; [32, 34]). One framework [32] comprised two overarching structural components: structural levels of impacts and timeframe.

The substructural components of the nine frameworks are described in various ways: principles of research capacity building [6, 17, 33]; activities [28, 29]; pathways to change [30]; objectives [34]; outcome indicator categories [31]; and impact domains [32]. All but one framework [6] present a series of outcome measures or indicators of research capacity. These metrics were categorized as partnerships and cross-sector collaborations; organizational research infrastructure; research training delivered and corresponding outcomes (trainee numbers, self-rated changes in knowledge and skill, among others); researcher career development and enrolment in tertiary-level research qualifications; research outputs; funding secured for research and RCB; research activity; new research roles and research leadership; information exchange events (symposia, showcases, among others); research capacity and gap analyses; evaluation and monitoring of programme impacts; and interest-holder engagement. Only one framework [17] included a metric that related to measuring health service users’ quality of life. Overall, there were many synergies and commonalities observed across the frameworks in terms of their development and their structural components, with evidence that later frameworks were informed by earlier frameworks and theories.

### Use of frameworks in research capacity evaluation practice

As of 18 September 2025, the nine frameworks were collectively cited in the peer-reviewed literature 889 times. Of the 889 citing articles, 481 cited Cooke’s [17] framework. In total, 37 out of the total 889 citing articles met the inclusion criteria; that is, they reported on evaluations of research capacity in health settings, using one or more of the frameworks. The 37 research capacity evaluations were conducted either as part of the process of planning RCB initiatives or measuring the outcomes or impacts of individual or multiple research capacity building initiatives (i.e. case studies, systematic or integrative reviews). The remaining citing articles (*n* = 852) did not meet the inclusion criteria. That is, they evaluated research capacity in academic or other non-health settings (see, for example, 41); the framework contextualized the study or the findings, but did not guide or inform the evaluation (see, for example, 42); the framework informed the evaluation of strategies (see, for example, 43); or informed an evaluation that was not published in a peer-reviewed journal (i.e. project reports, conference abstracts, theses).

The 37 articles reporting on evaluations of research capacity in health settings are summarized in Table 4. Most papers (*n* = 32) cited one framework; however, some articles (*n* = 5) cited two or more frameworks [44–48]. The most cited was Cooke’s earlier framework ([17], *n* = 31 articles), followed by Sarre’s framework ([33], *n* = 5 articles), Cooke’s adapted framework ([6], *n* = 4 articles), Bates’ revised framework ([29], *n* = 2 articles) and Bates’ original framework ([28], *n* = 1). Citing authors reported using (or for protocol articles, planning to use) the frameworks for one or more of the following purposes: data analysis or synthesis (*n* = 20 [45, 46, 48–65]); data collection (*n* = 19 [44, 47, 50, 51, 64–78]); programme development (*n* = 7 [50, 59, 68, 70, 74, 75, 79]); and/or to inform the interpretation of the results (*n* = 5 [45, 54, 67, 75, 80]).

**Table 4:** Use of frameworks in practice as reported in the peer-reviewed published literature

Lead author, year of publication Name of framework	Citing author, year	Programme name and/or research capacity evaluation context	Use or purpose(s) of framework in the study	Study design and methods of data collection and analysis	Key findings or learnings reported	Strengths and limitations of framework as reported by citing author
Cooke, 2005 Framework to evaluate research capacity building in health care [17]	Brown, 2021 [66]	•Rural-based research team of nutrition and dietetics academics within the University of Newcastle Department of Rural Health •Australia •High-income country	•Data collection: Cooke’s six principles of RCB [17] used to establish the data to be collected for the case study	•Longitudinal embedded quantitative case study design •Data sourced from public records, local observations and researcher records and documents •Outcomes extracted from sources included staffing levels, higher degree research enrolments, supervision and outcomes; honours student supervision and outcomes; internal and external grant outcomes; and peer-reviewed journal publications, along with journal impact factors •Data analysed descriptively	•Programme led to increased research staffing (full-time equivalent), higher degree by research enrolments and research student placements •Features of research team sustainability include the establishment of pilot projects, community links and an understanding of the local context to inform research activity, and networks with research expertise to support skill development and research outputs	Strengths •Nil reported Limitations •Nil reported
Cooke, 2005 Framework to evaluate research capacity building in health care [17]	Compaoré, 2021 [67]	•Global Maternal Sepsis Study (GLOSS) •16 countries in Africa and Latin America •Low- and middle-income countries	•Data collection: Cooke’s framework [17], along with three other frameworks, informed the focus group discussion guide •Discussion: Cooke’s framework [17] cited several times in the discussion section with authors interpreting, comparing and contrasting their findings according to Cooke’s	•Qualitative grounded theory study design •Data collected via focus groups and semi-structured interviews (*n* = 63) •Data analysed inductively and deductively using existing literature	•Study revealed unintended positive impacts in how participants approached their practice, demonstrating that participating in research and learning by doing can build capacity and improve practice •Challenges identified with involving novices in research and the need to involve local researchers early in the process to optimize capacity building opportunities	Strengths •The authors reflected on the relevance of Cooke’s [17] suggestion to consider research capacity building outcomes beyond dissemination of research results and consider impact on practice, patient and community-level outcomes Limitations •Nil reported
Cooke, 2005 Framework to evaluate research capacity building in health care [17]	Cooke, 2008 [68]	•Designated research team (DRT) •England •High-income country •Primary and community care developed by Trent Research and Development Support Unit (RDSU) •General practitioners, other practice staff, allied health, community nurses and pharmacists	•Programme development: Cooke’s framework [17] informed the development of the DRT programme •Data collection: Cooke’s six principles of RCB [17] informed the data collection tool	•Mixed-methods case study design •Set of process and outcome indicators for each of Cooke’s six principles [17] were identified a priori •Data collection form aligned with the principles and indicators and was used to assess teams’ research capacity (*n* = 6 teams) •Data sourced from documents, including meeting minutes and six-monthly written project reports, notes from discussions with the RDSU team, reflective team sessions and feedback from DRT leads •Data collated and consensus achieved through discussions	•Most teams developed research capacity, particularly in developing linkages and collaborations and skills domains •Authors caution that these outcomes may not be due to the DRT alone •Evaluating and reporting on traditional outcome indicators does not provide an accurate representation of the effectiveness of approaches to RCB; a fuller picture is necessary	Strengths •Cooke’s framework and the six principles [17] helped demonstrate the value of using a combination of less traditional process measures along with traditional so-called hard outcomes •The nontraditional measures captured local impact on practice and organizational development, and highlighted the importance of linkages and collaborations Limitations •Nil reported
Cooke, 2005 Framework to evaluate research capacity building in health care [17]	de Beer, 2024 [69]	•Investigating research capacity building needs •Saudi Arabia •High-income country •Tertiary care hospital (390 inpatient beds) •Clinical nurses	•Data collection: Cooke’s four structural levels and principles of RCB [17] informed the questionnaire items	•Quantitative descriptive cross-sectional study design •Data collected via a survey; survey questions organized according to six principles of RCB [17] •Data analysed via descriptive statistics and Spearman’s correlation	•Findings highlight top three research training needs, all relating to infrastructure principle •At least one-third of respondents were neutral on statements on building skills and confidence construct	Strengths •The framework was developed to evaluate the progress of RCB initiatives delivered in healthcare settings •Provides ground for assessing how and which RCB initiatives work and offers indicators to measure progress Limitations •Nil reported
Cooke, 2005 Framework to evaluate research capacity building in health care [17]	Flenady, 2022 [70]	•Research Ready Grant Program (RRGP) •Australia •High-income country •Partnership between a regional hospital and health service and a university rural clinical school •Multidisciplinary research teams	•Programme development: Cooke’s framework [17] informed the development of the RRGP •Data collection: Cooke’s six principles of RCB and four structural levels [17] informed the data collection tools, including the outcomes to be measured and the interview guide for RRGP participants and other stakeholders	•Exploratory concurrent mixed-methods study design •Data to be sourced from documents (attendance sheets, proposals and applications submitted; audit of research outputs, and review of quality improvement cycles) •Semi-structured interviews to be conducted with participants and other stakeholders (RRGP team members, mentors, guest presenters and future potential participants) •Survey of RRGP participants using Holden et al.’s Research Capacity and Culture (RCC) tool [82], which is informed by Cooke’s framework [17] •Qualitative data will be analysed thematically; quantitative data will be analysed descriptively •Cooke’s framework [17] will inform data analysis	•N/A: protocol paper	Strengths •Nil reported Limitations •Nil reported
Cooke, 2005 Framework to evaluate research capacity building in health care [17]	Fullam, 2018 [50]	•Research Excellence Across Clinical Healthcare (REACH) •Ireland •High-income country •28 metropolitan clinical services •Specialized and advanced nurses and midwives	•Programme development: Cooke’s framework [17] informed the development and implementation of REACH •Data collection: Cooke’s six principles of RCB and outcomes and example criteria [17] informed the outcomes measured •Data analysis or synthesis: Cooke’s six principles of RCB [17] used to analyse data	•Case study with 17 individuals and five small research groups supported by the REACH programme •Data sourced from documents, including annual reports, conference proceedings, manuscript reference lists, workshop and conference sign-in documents and evaluation forms •Data analysed deductively using Cooke’s six principles [17]	•Research outputs generated indicated that research capacity had been developed across the 17 individuals and five teams; with most participants having presented their work at conferences or published in journals within the 3-year evaluation period •Five individuals and one group had discontinued their research predominantly owing to unmanageable workloads	Strengths •Applying Cooke’s evaluative framework and principles [17] provided a useful structure to report the impacts of the RCB programme Limitations •Absence of valid impact indicators for health improvement and social impact
Cooke, 2005 Framework to evaluate research capacity building in health care [17]	Hedt-Gauthier, 2017 [51]	•Population health implementation and training (PHIT) partnership projects •Five African countries (Ghana, Mozambique, Rwanda, Tanzania and Zambia) •Low- and middle-income countries	•Data collection: Cooke’s six principles of RCB [17] informed the collection of data from RCB activity leads from each country •Data analysis or synthesis: Cooke’s six principles and four structural levels [17] used to synthesize the project activities specific to RCB	•Case studies (*n* = 5) •Data collected from representatives from each RCB programme using a data collection form/survey to capture quantitative and brief qualitative data •RCB activities and priorities were informed by Cooke’s six principles [17] and were summarized quantitatively •Programme metrics summarized across the four structural levels of influence; five key activities were mapped to the six principles of RCB [17]	•Six key lessons identified across the case studies: funders of RCB must provide long-term and flexible support; RCB strategies must comprise a continuum of activities; focus on strengthening existing research institutions; research should align with health programme implementation to optimize RCB; mentorship is essential to RCB, but creative approaches may be required; and measuring and monitoring the impacts of RCB is essential to ensuring effectiveness of strategies	Strengths •Cooke’s framework [17] provided a cohesive mechanism to synthesize activities across different projects Limitations •Nil reported
Cooke, 2005 Framework to evaluate research capacity building in health care [17]	Hilder, 2020 [71]	•Supported research funding initiative •Australia •High-income country •Allied health professionals working at a health service (*n* ~880)	•Data collection: Cooke’s four structural levels [17] informed the interview questions	•Qualitative study informed by a realist evaluation •Data collected via semi-structured interviews (*n* = 10) •Data analysed thematically using a deductive approach	•A funding initiative led to increased research opportunities, research outputs, changes to practice and improved research capacity and culture •Outcomes were influenced by contextual factors, including supportive managers/leaders and research infrastructure within the health service •Mechanisms to achieve the outcomes included researcher motivation and access to suitable clinical backfill	Strengths •Nil reported Limitations •Nil reported
Cooke, 2005 Framework to evaluate research capacity building in health care [17]	Jeffs, 2018 [72]	•Collaborative practice-based RCB educational programme •Canada •High-income country •Urban teaching hospital •Nurses and other health disciplines	•Data collection: Cooke’s framework [17] and other literature informed the interview questions and probes •Unclear which elements of Cooke’s framework [17] were used	•Descriptive qualitative design •Data collected via interviews and focus groups with programme participants (*n* = 12) •Data analysed thematically using an inductive approach	•Programme provided opportunities to engage in collaborative practice-based research •Support from mentorship team and managers identified as important •Challenges included the need for protected time and backfill, support from research personnel and funds for knowledge translation activities	Strengths •Adds to the RCB literature, which the authors describe is primarily based on survey studies Limitations •Nil reported
Cooke, 2005 Framework to evaluate research capacity building in health care [17]	Khan, 2021 [52]	•Collaboration for Leadership in Applied Health Research and Care North West Coast (CLAHRC-NWC) •England •High-income country	•Data analysis or synthesis: Cooke’s six principles of RCB and four structural levels [17] informed the analysis process; qualitative data were coded and results presented according to the six principles of RCB [17]	•Qualitative study design •Data collected via semi-structured interviews, focus groups and workshops (*n* = 131) •Data analysed deductively using Cooke’s six principles [17]	•Learning through experience helped build knowledge, skills and confidence in a diverse group of health NHS staff and contributed to the development of organizational research culture and research networks •Key enablers were research resources and infrastructure	Strengths •Cooke’s principles [17] provided a useful lens to explore the impacts of RCB initiatives beyond the traditional focus on training delivered and outputs achieved (e.g. formal qualifications, grants and publications •By exploring processes involved in RCB, the authors identified modest changes in organizational culture, research experience, knowledge, and skills and examples of research collaboration and impacts Limitations •Nil reported
Cooke, 2005 Framework to evaluate research capacity building in health care [17]	King, 2022 [53]	•The Turning Ideas into Proposals research training programme •Australia •High-income country •Rural and regional public health service settings in western Victoria •Point-of-care allied health professions	•Data analysis or synthesis: Cooke’s four structural levels [17] used with other literature to develop a coding framework for thematic analysis of interview data	•Sequential mixed-methods study design •Data collected via a survey, 23-item Evidence-Based Practice (EBP)-Knowledge, Attitudes and Practice, administered at baseline (pretraining) and 3 months after •Semi-structured interviews 3 and 16 months post training •Quantitative data analysed descriptively and using analysis of variance (ANOVA) and Wilcoxon signed-rank test •Qualitative data analysed using a team-based inductive thematic framework approach and a deductive approach using Cooke’s framework [17]	•Surveys showed no significant improvements detected across EBP knowledge, practice or attitudes (small sample) •Interview participants valued practical, applied, mentored nature of training, built confidence and basic research skills. Programme outcomes ranged from ceased project initiatives to finalized study protocols; organizational enablers and barriers were highlighted •Ripple effects on organizations (e.g. participants contributed to organizational research capacity through networking, sharing learnings, inspiring colleagues, among others) •The interrelatedness of RCB strategies and levels of influence were reinforced	Strengths •Framework enabled the exploration of unquantifiable benefits of releasing the potential of emerging researchers and organizations Limitations •Nil reported
Cooke, 2005 Framework to evaluate research capacity building in health care [17]	Lee, 2023 [73]	•Investigating research capacity and productivity of Canadian sports chiropractors •Canada •High-income country •Sports chiropractors	•Data collection: Cooke’s six principles of RCB [17] used to modify an existing survey tool to measure the research capacity and resource survey	•Cross-sectional quantitative study design •Data collected via a survey of sports chiropractors, and •Scoping review of sports chiropractor-focused research outputs •Survey data analysed descriptively; scoping review data extracted and analysed numerically, thematically and descriptively	•Sports chiropractors tend to engage in research on a part-time basis, with few holding PhD qualifications •Many sports chiropractors provide junior clinicians with research mentoring	Strengths •Cooke’s framework [17] outlines the importance of interventions at the four structural levels of influence •Framework encouraged the collection of data from multiple sources (self-report data and published literature) Limitations •Nil reported
Cooke, 2005 Framework to evaluate research capacity building in health care [17] Sarre, 2009 Indicators for measuring research capacity development in primary care organizations [33] Cooke, 2021 Research Capacity Development for impact (RCDi) framework [6]	Lee, 2024 [45]	•Exploring the challenges and opportunities for research capacity development •Canada •High-income country •Sports chiropractors	•Data analysis or synthesis: Cooke’s six principles of RCB [17] guided coding of the interview data •Discussion: Cooke’s structural levels [6, 17] and Sarre’s framework [33] cited frequently in interpretation of study findings	•Qualitative descriptive study design •Data collected via semi-structured interviews with chiropractic researchers and focus groups with organizational leaders in sports chiropractic •Data analysed using interpretive content analysis approach	•Findings highlight challenges and opportunities for RCB in sports chiropractic in Canada •The following actions are needed to build research capacity in this group: securing sustainable funding, expanding research affiliations and partnerships, higher degree research pathways, dedicated research time, aligning the research agenda with stakeholder priorities, establishing a research centre and formalizing research networks	Strengths •Cooke’s frameworks [6, 17] aligned well with the study findings and therefore could be used to inform RCB strategies for the chiropractic profession Limitations •Nil reported
Cooke, 2005 Framework to evaluate research capacity building in health care [17]	Luckson, 2018 [54]	•Exploring research culture at two hospitals •England •High-income country •Metropolitan •One research-focused hospital introduced RCB initiatives in preceding decade and one non-research-focused hospital •Nurses and allied health professionals	•Data analysis or synthesis: Cooke’s six principles and RCB and three (of four) structural levels [17] informed the data analysis •Discussion: references to Cooke’s four structural levels and six principles of RCB [17] in the discussion to present and help interpret the findings of the study •Survey (RCC tool [82]) was informed by Cooke [17]	•Mixed-methods study design •Data collected via a survey (Research Capacity and Culture tool; *n* = 224), and •Focus groups (*n* = 14) and individual semi-structured interviews (*n* = 5) •Data analysed using a framework approach in reference to Cooke’s structural levels [17]	•Efforts to build research capacity must focus on the team level and a whole-organization view to develop a strong research environment and culture •More barriers to research were identified in the research-focused hospital •Cooke’s six principles [17] with a new communication (within and across organizations) domain were suggested as a useful framework to improve organizational research culture	Strengths •Using Cooke’s framework [17] aided the evaluation of the whole-system approach to RCB Limitations •Authors suggested that Cooke’s framework should include communication along with the other six principles
Cooke, 2005 Framework to evaluate research capacity building in health care [17]	McGuire, 2020 [55]	•The Family Medicine Specialty Training Programme (FMSTP) •Lesotho (South Africa) •Middle-income country •Accredited postgraduate medical education programme •Academic partnership with the Lesotho Ministry of Health and the Lesotho-Boston Health Alliance •Family physicians	•Data analysis or synthesis: Cooke’s six principles of RCB [17] informed the data analysis; results organized and reported according to Cooke’s six principles [17]	•Longitudinal mixed-methods study design •Data collected via Likert-scale surveys measuring trainee research confidence; written evaluations by trainees, peers, programme faculty and administrators, and •Semi-structured interviews •Survey data analysed using Friedman and sign tests •Interview and written data analysed thematically via a mixed inductive–deductive approach using Cooke’s framework [17]	•The FMSTP improved trainees’ research skills and confidence and supported the conduct of research that was close to practice •Peer mentorship supported flexible learning •Work is required to address the remaining Cooke’s four principles [17]: linkages and collaborations; actionable dissemination; continuity and sustainability; and research infrastructure	Strengths •Cooke’s framework [17] focuses on process domains that are relevant to novice researchers and can be measured more proximally •It also focuses on outcome domains that consider the community health impact of research, which are especially relevant to family medicine •Cooke’s framework [17] helps to explain how outcomes in one area/principle of RCB interconnect with another Limitations •Inadequate progress and gaps were noted in some of Cooke’s RCB principles, which the authors attribute to the timing of the data collection (mid-way through the program) and content of the interview guide (questions about linkages and collaboration were not asked)
Cooke, 2005 Framework to evaluate research capacity building in health care [17]	Misso, 2016 [74]	•Protocol for the co-design, development, implementation, evaluation, refinement and scale-up of the Clinical Research Engagement and Leadership Capacity Building Program •Australia •High-income country •Large health service and academic health science centre	•Programme development: Cooke’s framework [17] will inform the development and implementation of the clinical research engagement and leadership capacity building programme •Data collection: Cooke’s six principles will inform the outcomes measured at Cooke’s four structural (i.e. individual, team, organizational and external engagement) levels [17]	•Longitudinal mixed-methods study design •Data to be collected via surveys informed by Cooke’s outcomes and indicators at the four structural levels [17] •Interviews with clinical and research programme leaders •Research database searches for research and documents published as evidence of linkages, partnerships and collaborations, dissemination and impact, and •Local health service database search of research projects and funding •Quantitative data will be analysed descriptively and using regression techniques comparing pre- and post-data •Qualitative data will be analysed thematically	•N/A: protocol paper	Strengths •Nil reported Limitations •Nil reported
Cooke, 2005 Framework to evaluate research capacity building in health care [17] Sarre, 2009 Indicators for measuring research capacity development in primary care organizations [33]	Mosedale, 2022 [46]	•Research Translation Program (RTP) •Australia •High-income country •Research funding programme to support collaborative research	•Data analysis or synthesis: Cooke’s [17] and Sarre’s [33] principles of RCB used along with the Canadian Academy of Health Sciences’ framework to analyse the data	•Longitudinal mixed-methods study design (2011–2019) •Data sourced from documents (application guidelines, grant submissions and project completion reports) •Documentary data analysed deductively against Cooke’s six principles [17] •Collaborations between clinicians and academics were analysed using social network analysis •Sarre’s framework [33] informed thematic and social network analyses	•RTP grant programme and collaborative research contributed to high-quality research outputs: peer reviewed publications, conference presentations, media outputs, educational resources, theses and reports •Changes to policy and practice through the implementation of guidelines in the local setting were apparent •Social network analysis showed new collaborations across the health system via the RTP •More research needed to understand the pathway by which short-term outcomes can lead to longer-term impacts	Strengths •Cooke’s framework [17] facilitated the evaluation of the RTP beyond traditional markers of research capacity Limitations •Nil reported
Cooke, 2005 Framework to evaluate research capacity building in health care [17]	Mugabo, 2015 [56]	•Research capacity strengthening training activities outside formal academic programmes •Countries in sub-Saharan Africa •Low- and middle-income countries	•Data analysis or synthesis: Cooke’s six principles of RCB and example criteria [17] used to assess research training effectiveness and present the results	•Systematic review •Systematic search for articles reporting on health RCB initiatives delivered in non-academic settings in sub-Saharan Africa (*n* = 14 articles) •Data related to the effectiveness of research training programmes extracted from included articles •Outcomes data analysed deductively according to 19 of Cooke’s 92 outcome measures/indicators of RCB	•Conduct of evaluations and use of evaluation frameworks varied between short- and long-term models •Institutional support, increased funds and dedicated time for research activities are critical factors leading to development of successful programmes •Replication of successful models relies on robust evaluation methods and programme documentation in peer-reviewed literature	Strengths •Cooke’s framework [17] provided a comprehensive description of indicators for individual training in RCB Limitations •Nil reported
Cooke, 2005 Framework to evaluate research capacity building in health care [17]	Njie-Carr, 2012 [75]	•RCB Programme for Reach Out (human immunodeficiency virus [HIV] prevention, support, treatment and care programme) •Uganda •Low-income country •Research training for Reach Out employees	•Programme development: Cooke’s framework [17] provided the conceptual framework for the planning, design and implementation of RCB programme •Data collection: three data collection tools reflect the principles of Cooke’s framework [17] •Discussion: references to Cooke’s principles of RCB [17] in the discussion •Elements of framework used are not stated	•Descriptive mixed-methods cross-sectional evaluation study design •Data collected via surveys of participants at three timepoints (initial situational analysis; interim evaluation; final evaluation), generating qualitative and quantitative data •Qualitative data analysed using a content analysis approach; quantitative data analysed descriptively	•Situational analysis led to focused RCB training programme being successfully designed and implemented •Trainees gained concrete, applicable skills in research methods, and reported increased knowledge and confidence •Barriers and facilitators to RCB training and engagement in research post-training identified (time, among others)	Strengths •Cooke’s framework [17] and the principles of building skills and confidence, developing partnerships, promoting close to practice research, and sustainability were useful in guiding programme development and evaluation Limitations •Nil reported
Cooke, 2005 Framework to evaluate research capacity building in health care [17] Sarre, 2009 Indicators for measuring research capacity development in primary care organizations [33]	Nott, 2024 [48]	•Scoping review of collaborations between health services and educational institutions that aim to build research capacity •Studies from Australia, the United States, Canada, Rwanda, Africa, New Zealand, Brazil and Asia Pacific •Low-, middle- and high-income countries •Collaborations with the explicit intention of building research capacity in health service employees	•Data analysis or synthesis: three of Cooke’s four structural levels [17] and Sarre’s [33] frameworks were used in the analysis of textual data extracted from the papers and to present the results	•Systematic scoping review •Systematic search for articles reporting on partnerships and collaborations to improve research capacity in health settings (*n* = 61 articles) •Data related to research capacity building activities and outcomes were extracted from the included articles •Outcomes data analysed via a deductive content analysis approach according to three of Cooke’s [17] and Sarre’s [28] structural levels	•Models and approaches to partnerships must consider the values and needs and capabilities of each of the partner organizations •Clear alignment between partners’ priorities and intended outcomes for the partnerships to be successful and sustained •Outcomes were identified at the three structural levels of influence; however, these outcomes could not be linked to specific partnership models or approaches •Papers included in the review did not report changes/outcomes at the supra-organizational level	Strengths •Viewing collaboration models and outcomes according to Cooke’s [17] and Sarre’s [33] structural levels of RCB influence helped reveal enablers and barriers to research capacity building, the principles and practicalities of effective partnerships Limitations •Nil reported
Cooke, 2005 Framework to evaluate research capacity building in health care [17]	Payne, 2012 [57]	•Cancer Experiences Collaborative (CECo) •England/United Kingdom •High-income country •Research partnership organization (universities, hospices and cancer centres, charity organization and service user representatives)	•Data analysis or synthesis: outcomes in CECo reports were categorized according to Cooke’s six principles and some of the example criteria [17]	•Case study design •Data pulled from annual and final reports, and other CECo records and documents •Data analysed via a deductive categorization process	•CECo programme led to increased research capacity of United Kingdom researchers •Quality and volume of collaborative palliative care have increased •CECo was also deemed to have influenced research, policy and practice within and beyond the United Kingdom	Strengths •Cooke’s six principles of RCB [17] were useful in assessing the process as well as the outcomes of CECo’s RCB activity •Some principles were less developed than others; it takes longer to demonstrate the impact of the investment in newer research Limitations •Nil reported
Cooke, 2005 Framework to evaluate research capacity building in health care [17]	Perez Liz, 2024 [58]	•Autism Global Panel (AGP) •Northern Mexico •Middle-income country •An autism research collaborative to build capacity for autism research	•Data analysis or synthesis: Cooke’s four structural levels, six principles of RCB, and example criteria [17] were applied to the list of organization leaders’ AGP priorities to be addressed by the RCB effort	•Case study design •Data collected via oral narratives from two founding members of the AGP, and input via the local research team •Documentary data from project and administrative records, written communication between teams •Data categorized to the relevant criteria and summarized descriptively	•Over time, progress had been made across all six RCB principles [17] •The two principles with the highest rate of achievement were linkages, collaborations and partnerships; and infrastructure	Strengths •Cooke’s framework [17] provided the flexibility to measure intermediate/proximal outcomes of the RCB processes Limitations •Research is needed to explore the link between intermediate achievements and longer-term outcomes •Framework did not enable weighting of the criteria or structural levels of RCB
Cooke, 2005 Framework to evaluate research capacity building in health care [17]	Quilliam, 2023 [76]	•Identifying the design and implementation characteristics of current research training for rural health professionals •Australia •High-income country •Research training for rural health professionals in Victoria	•Data collection: several of Cooke’s principles of RCB framework [17] informed interview guide, questions and prompts	•Qualitative descriptive study design •Data collected via semi-structured interviews with key informants (*n* = 20) with extensive knowledge of research education and training in rural health services •Data analysed using an inductive thematic approach and mapped to the Consolidated Framework for Implementation Research domains	•Lack of tailored and strategic coordination for rural context •Tensions between research opportunities and clinical practice inhibit rural health professional engagement in research education/training •Coordinated approach with experiential learning, flexible modes of delivery and use of research networks that provide training opportunities in a timely manner is needed	Strengths •Use of Cooke’s framework [17] ensured interrogation of context-specific concepts during interviews Limitations •Nil reported
Cooke, 2005 Framework to evaluate research capacity building in health care [17]	Schmidt, 2014 [59]	•Rural Research Capacity Building Program (RRCBP) •Australia •High-income country •Rural and remote health services in New South Wales •Nursing, allied health and other health workers	•Programme development: Cooke’s framework [17] informed the RRCBP •Data analysis or synthesis: Cooke’s four structural levels [17] informed classification of the reasons for withdrawal from the RRCBP	•Retrospective case–control quantitative study design •Data collected from documents (researchers’ files and programme data) related to programme participants from 2006 to 2010 (*n* = 104) •Data analysed using exact logistic regression, chi-squared test and Fisher’s exact test •Reasons for withdrawal and participant characteristics were categorized according to Cooke’s four structural levels of influence [17]	•Programme attrition rate of 29% •Withdrawals were linked to individual (e.g. lack of passion, workload, personal issues), organizational (e.g. limited managerial support, no backfill) and supra-organizational factors (e.g. leaving the organization) •Attrition was closely tied to the availability of timely and appropriate support, particularly those working in professional or geographic isolation	Strengths •Nil reported Limitations •Nil reported
Cooke, 2005 Framework to evaluate research capacity building in health care [17]	Schmidt, 2016 [80]	•Research Capacity Building Program •Australia (rural and remote areas) •High-income country •Primary healthcare organizations in partnership with the Centre for Research Excellence in Rural and Remote Primary Health Care Research (CRE)	•Discussion: Cooke’s six principles of RCB and several example criteria [17] were cited in the discussion to interpret the results of the evaluation and to examine the elements, strengths and weaknesses of the RCB programme	•Cross-sectional quantitative study design •Data collected via a survey •Self-reported research experienced was assessed using the research spider tool administered after an introductory research methods workshop and reassessed at programme completion (2 years later) •Data analysed descriptively and using Wilcoxon signed-rank test	•Trainees reported significant gains in research skills and expressed interest in continuing research •Research projects matched organizational goals •Trainees’ outputs varied at programme completion •Modular programme structure fostered local peer support networks and cross-site collaboration •RCB programme will likely lead to increased research capacity with respect to several of Cooke’s principles •Outcomes related to the principles of RCB less evident through the programme were: research continuity and sustainability; enhanced research culture; linkages and partnerships; and research infrastructure	Strengths •Nil reported Limitations •Nil reported
Cooke, 2005 Framework to evaluate research capacity building in health care [17]	Schmidt, 2022 [60]	•Rural Research Capacity Building Program (RRCBP) •Australia •High-income country •Rural and remote health services in New South Wales •Nursing, allied health and other health professionals	•Data analysis or synthesis: three of Cooke’s four structural levels [17] informed the second (deductive)-phase analysis of qualitative data	•Qualitative study underpinned by critical realism •Data collected via interviews and focus groups (*n* = 22) •Data analysed via initial inductive thematic analysis and subsequent deductive analysis using Cooke’s structural levels of influence [17], excluding supra-organizational level	•Some key outcomes for individuals included new research skills, improved work performance and employability •Some individuals with research experience felt discontent and perceived a lack of organizational support for research •Some organizational outcomes included raised research profile, increased local and relevant research and evaluation activity, and staff retention	Strengths •Nil reported Limitations •Nil reported
Cooke, 2005 Framework to evaluate research capacity building in health care [17]	Tadele, 2023 [61]	•Needs assessment undertaken to identify capacity for conducting abortion-related research •Ethiopia •Low-income country •Professional individuals involved in abortion-related research	•Data analysis or synthesis: Cooke’s framework informed the deductive analysis of qualitative survey responses; results presented according to Cooke’s six principles [17]	•Cross-sectional mixed-methods study design •Data collected via a survey of researchers and stakeholders (quantitative and qualitative questions), and •Focus group with a smaller sample •Quantitative survey data analysed descriptively; qualitative survey data and focus group data analysed deductively using Cooke’s framework [17]	•Inadequate research skills and poor linkages and collaboration between health practitioners and academic researchers were key barriers to the conduct of abortion research •Limited research dissemination platforms and funding also limit the impact and sustainability of research in Ethiopia	Strengths •Nil reported Limitations •Nil reported
Cooke, 2005 Framework to evaluate research capacity building in health care [17] Cooke, 2021 Research Capacity Development for impact (RCDi) framework [6]	Tavares, 2025 [47]	•Examine infrastructure to enable research engagement •Canada •High-income country •Ontario (metropolitan) •Paramedics	•Data collection: Cooke’s principles of RCB [17] and RCDi [6] informed the interview guide	•Qualitative study design •Data collected via semi-structured interviews (*n* = 24) •Data analysed via reflexive thematic analysis informed in part by Cooke’s frameworks and principles [6, 17]	•Structural and cultural inadequacies challenge existing strategies to build research capacity in the paramedicine profession •Without overcoming structural and cultural barriers, the paramedicine profession is limited in its ability to effectively generate and translate research knowledge in practice •Actions required to build research capacity in paramedicine include the implementation of robust infrastructure, equitable access, interdisciplinary collaboration and embedding research in practice	Strengths •Cooke’s frameworks [6, 17] provided a conceptual structure upon which to explore the research infrastructure and develop recommendations Limitations •Nil reported
Cooke, 2005 Framework to evaluate research capacity building in health care [17]	Twelvetree, 2024 [79]	•Postdoctoral clinical research support programme •England •High-income country •Metropolitan	•Programme development: Cooke’s six principles of RCB and four structural levels [17] defined, informed and helped describe the approach to building postdoctoral clinician–researcher skills	•Reflective piece/case study •No data collection or analysis methods described •Author describes the approach to developing research skills according to Cooke’s structural levels and principles of RCB [17]	•Factors most integral to building research capacity in the nursing, midwifery and allied health professions are strategic senior manager support, targeted support for groups of researchers, and realistic and often longer timeframes for research activity •A “whole of organization” approach to developing postdoctoral clinical academic skills that enables flexible action learning contributes to individual and organizational RCB	Strengths •Cooke’s framework [17] provides a clear structure Limitations •Contextualizing proposed change and using action learning to develop a research culture are important additional elements to consider when developing research capacity
Cooke, 2005 Framework to evaluate research capacity building in health care [17]	Van Rensburg 2017 [62]	•Research capacity development programme for novice researchers •Metropolitan location •South Africa •Middle-income country •Nursing educators not attached to education institutions	•Data analysis or synthesis: Cooke’s principles of RCB [17] (modified version of Cooke’s framework developed by Kahwa, et al. [83; p. 23]) informed deductive data analysis •Results reported according to Cooke’s principles [17]	•Exploratory case study design •Data sourced from documents, including annual reports (*n* = 6), reflective activities (*n* = 3) and conference presentations (*n* = 3) •Data analysed deductively using Cooke’s (modified) framework [17, 83] as the foundation, following iterative process	•Programme success lies in skills and confidence gained and contribution made in addressing education practice problems •Strong partnerships developed through sharing project responsibilities •Programme leaders supported work to be implemented and shared •Programme continuity and sustainability remain a challenge	Strengths •Cooke’s modified integrated framework [17, 83] enabled exploration of mechanisms of RCB •Framework addresses important programme aspects (e.g. sustainability and continuity) •Analysis based on an established conceptual framework helped reduce bias in programme evaluation Limitations •Nil reported
Cooke, 2005 Framework to evaluate research capacity building in health care [17]	Wong Shee, 2022 [77]	•Exploration of key informants’ knowledge of the drivers and challenges for RCB in rural areas in the local setting •Australia •High-income country •Rural public health services health-related research or health professional education in Victoria	•Data collection: Cooke’s six principles of RCB [17] informed interview guide and questions	•Qualitative study design •Data collected via using semi-structured interviews •Data analysed thematically and deductively using the Consolidated Framework for Implementation Research for understanding implementation context	•A diverse range of factors influence RCB in rural health settings, including RCB intervention-specific factors, health service factors, policies and other factors external to health services, individual health professional factors and processes for implementing RCB strategies •Addressing contextual factors characteristic of rural health settings will promote positive RCB outcomes	Strengths •Cooke’s framework [17] focuses on exploring mechanisms and outcomes of RCB Limitations •Cooke’s framework [17] does not explore the influence of context on the implementation of RCB initiatives
Bates, 2006 KATH tool [28] Bates, 2011 Indicators of sustainable capacity building for health research [29]	Cole, 2016 [44]	•Health Research Capacity Strengthening Initiative (HRCSI) •Malawi •National research funding program	•Data collection: Bates’ KATH tool [28] and four phases of RCB progress influenced the structure of the interview guide, and Bates’ indicators [29] informed interview questions	•Mixed-methods review/case study design •Data sourced from documents on websites, HRCSI staff (proposals, national research agenda, evaluation reports, conference programmes, grant documents; *n* = 21), and •Interviews conducted with funders, research managers/governors, producers and users of research (*n* = 30) •Data analysed deductively according to the four phases of progress	•The HRCSI produced a range of outcomes according to the perspectives and influences of various actors engaged in the programme •Outcomes included completion of a priority setting exercise, development of institutional research capacity, facilitated knowledge sharing and dissemination, and established some sustainability	Strengths •Using the Bates’ [28, 29] frameworks, the case study was able to demonstrate the benefits of starting small and expanding gradually, to build trust and relationships Limitations •Nil reported
Sarre, 2009 Indicators for measuring research capacity development in primary care organizations [33]	Probst, 2015 [78]	•Audit of research capacity •United Kingdom •Radiography therapy workforce	•Data collection: Sarre’s [33] nine principles were adapted to create a survey tool	•Quantitative study design •Data collected via two survey tools for two separate groups: radiotherapy services managers (*n* = 45) and research radiographers (*n* = 30) •Data analysed descriptively •Results reported according to Sarre’s [33] nine principles	•Several short- and longer-term strategies are needed to promote the research culture and capacity of the therapy radiography workforce •Strategies to promote research capacity include using existing research infrastructure, implementing a research coordinator or lead to support and motivate others, the development of networks and linkages, and a research strategy linked to organizational priorities	Strengths •Nil reported Limitations •Nil reported
Sarre, 2009 Indicators for measuring research capacity development in primary care organizations [33]	Ruco, 2021 [65]	•Practice-based research and innovation (PBRI) strategy •Canada •High-income country •Sunnybrook multisite academic health science centre •Point-of-care health professionals	•Data collection: Sarre’s principles were adapted to inform the metrics that were embedded in the evaluation to measure growth and impact •Data analysis or synthesis: Sarre’s [33] principles (five out of nine) were used to analyse the data and present the results	•Case study/programme evaluation •Data collected at multiple timepoints for each activity or programme (e.g. pre- and post-program) and cross-sectional data collection •Data collected from program/activity leads and individuals •Data summarized and presented descriptively •Results reported according to five of Sarre’s nine principles	•Collaborative leadership leveraged linkages and partnerships, and the recognition of academic contributions of health professionals contributed to the PBRI’s success •PBRI led to increased RCB opportunities for health professionals •Organizations should adopt an RCB evaluation framework that aligns with their context, with a focus on infrastructure, linkages and partnerships, research and innovation activity, culture, continuity and sustainability	Strengths •Sarre’s [33] framework chosen for its alignment with the context within which the PBRI strategy was implemented •Sarre’s [33] framework has a strong focus on capturing the impact of relationships, collaborations and linkages – indicators that are perceived to be more important than traditional research outputs Limitations •Nil reported
Bates, 2011 Indicators of sustainable capacity building for health research [29]	Bates, 2015 [49]	•Case studies of four programmes: KATH Ghanaian Teaching Hospital; Kenyan nongovernmental organization (NGO); Malawian Research Unit Trust; Democratic Republic of the Congo Research and Training •Low- and middle-income countries	•Data analysis or synthesis: Bates’ phases of RCB progress [29] used to systematically compare the four case studies	•Case studies •Informed by authors’ experience and knowledge of the cases •No description of data collection or analysis methods	•A structured approach to designing and evaluating RCB strategies is needed •Evaluations must be robust, well informed and contextually appropriate, and consider the outcomes and indicators that can be measured •Evaluations with a defined change pathway (theory of change) and engagement with stakeholders in all stages produce meaningful outcomes	Strengths •Nil reported Limitations •Nil reported
Cooke, 2021 Research Capacity Development for impact (RCDi) framework [6]	McGuire, 2025 [63]	•Review of virtual health research capacity strengthening (HRCS) programmes •Low- and middle-income countries •Clinicians	•Data analysis or synthesis: Cooke’s modified RCDi framework [6] was used to analyse the data and present results	•Integrative review •Articles searched and retrieved using a systematic approach •Data extracted from the included articles and analysed deductively using Cooke’s RCDi modified framework [6] •The Mixed Methods Appraisal Tool was used to assess programme evaluations	•Across the sample of HRCS programmes, skills, confidence building and sustainability were the most discussed impacts •Interconnectedness among the framework domains suggests advances across the domains were synergistic	Strengths •The nuanced, multilevel and intersectional approach to evaluating HRCS programme impacts •Inclusion of both process and outcome measures •Tool identifies actionable insights •Framework can guide improved programme design and novel evaluation strategies Limitations •Cooke’s framework [6] was developed from HRCS experience in a high-income context, and there was a gap in its ability to assess impacts on equity •Authors modified the framework to include an equity output/outcome domain
Cooke, 2021 Research Capacity Development for impact (RCDi) framework [6]	Wolstenholme, 2025 [64]	•Exploring stakeholders’ view of the factors influencing successful implementation of community-based research roles •England •High-income country •Nurses and midwives working in community setting	•Data collection: Cooke’s seven principles of RCDi [6] informed the interview guide •Data analysis or synthesis: data analysed and reported according to Cooke’s seven principles of RCDi [6]	•Sequential mixed-methods exploratory study design •Data collected via a survey (*n* = 64), and •Interviews (*n* = 19) •Data analysed using the framework method and informed by Cooke’s seven principles and criteria [6] •Findings reported according to Cooke’s principles [6]	•Key enabling features of research roles reflect Cooke’s seven principles and are underpinned by trust and relationships •All seven principles must be considered in the development and implementation of nursing and midwifery research roles •The seven principles can be addressed over time as the roles move from initiation to continued growth through to ambition	Strengths •Considering the RCDi framework [6] in a temporal sense facilitates planning and implementing nursing and midwifery research roles in community settings Limitations •Nil reported

The vast majority of citing papers used some, but not all, of the framework components. Typically, the overarching or substructural components were used in the evaluations, with only four articles using the outcomes, indicators or criteria for RCB as outlined by Cooke’s [17] framework [50, 56–58]. Some of the citing articles demonstrated very minimal use of the frameworks [67, 71, 72, 81]. Some authors were explicit in their rationale for and application of the cited evaluation framework (e.g. [58]), where for others, the application of the framework was implied and not specified (e.g. [72]). Most evaluations partially or fully comprised of self-reported data generated via surveys of, or individual interviews or focus groups with programme participants and/or leads. One evaluation was akin to a reflective, author-informed case study [79]. Nine evaluations included data drawn from a range of documents and reports sourced from public and organizational records [46, 50, 57–59, 62, 66, 70, 74].

### Limitations and strengths of research capacity evaluation frameworks

#### Framework development

Six of the nine framework study authors reported limitations related to the development of their published frameworks (Table 2). These limitations related to biases introduced during the framework development [29–31]; and a lack of focus on relationships, transferability, relevance in and value to other contexts [17, 28, 31, 33]. All framework study authors described strengths of their frameworks (Table 2). These strengths were the flexibility, adaptability and transferability of indicators for evaluating research capacity in different contexts [29, 30, 32]; affording institutions in developing countries the autonomy to set their own RCB priorities and evaluate progress accordingly [28]; combining process and outcome indicators [17]; guiding research–practice partnerships [6]; aligning health service organizational strategic plans and RCB strategies [34]; providing a comprehensive list of the most common indicators of research capacity [31]; lending a structured approach to programme development with a focus on medium- and longer-term outcomes [32]; and building on Cooke’s framework [17] contributing three additional domains [33].

#### Framework application

Overall, 7 of the 37 citing articles described limitations associated with using the frameworks in practice (Table 4). One citing author referred to the variable time taken to achieve different outcomes, meaning evidence of some principles was not captured [55]. One citing author noted the evaluation of individual-level outcomes (e.g. knowledge and satisfaction) often relies on participant self-selection and self-reporting, potentially skewing towards favourable outcomes [72]. Others noted the evaluation frameworks do not identify higher priority RCB principles or outcomes [45], nor the weighting of the criteria and structural levels of influence [58]. Some citing authors pointed to missing components in the frameworks, including indicators of health improvement and social impact [50], communication [54], and equity [63]. The inadequate emphasis on context [77] and implications for real-world practice and outcomes were also identified limitations [58].

Twenty-seven citing authors identified strengths associated with using the framework/s in their evaluation. Authors who cited Cooke’s framework [17] highlighted its applicability to healthcare settings [69]; ability to facilitate a nuanced analysis of RCB mechanisms [62]; focus on outcomes beyond traditional research outputs [46, 52, 53, 65, 67, 68] including proximal measures of progress [55, 58, 69, 77]; composition of a diverse range of indicators for measuring outcomes and impacts [56]; context-specific concepts [76] that encourage novel evaluation approaches [55]; and different methods of and sources for data collection, contributing diversity to the RCB literature [72, 73]. Several citing authors noted that frameworks and principles lend a systematic approach to exploring research infrastructure [47], and synthesising and reporting a range of outcomes [50, 51, 75]; thereby demonstrating how RCB principles interrelate with others [55], and reducing bias in evaluation processes [62].

Authors identified the benefit of Cooke’s earlier [17] and later [6] frameworks in informing RCB initiatives and strategies [64]; specifically, by reinforcing the importance of building research capacity at multiple levels to promote research culture at the system level [45, 54, 73], guiding programme improvements and informing actionable insights and recommendations [47, 55, 79]. In combination with Sarre’s framework [33], Cooke’s framework [17] helped reveal barriers and enablers to RCB and the principles and practicalities of establishing effective partnerships [48]. Bates’ KATH Tool [28] facilitated the demonstration of the benefits of starting small and expanding gradually to build trust and relationships [44].

## Discussion

To our knowledge, this is the first methodological review of peer-reviewed, published frameworks designed to evaluate research capacity in health settings. Cooke’s framework [17] was the earliest to guide the evaluation of research capacity in health settings, and demonstrates sustained utility as evidenced by continual citations. The frequency of citations may in part be due to its publication in an open-access journal; however, it also suggests that the framework resonates with RCB researchers and programme leads across different contexts. A further eight research capacity evaluation frameworks have since been published. Commonalities and synergies were observed across all nine frameworks, with evidence that each built on earlier theories of individual and organizational capacity building. Despite the high number of collective citations of the nine frameworks, a comparatively small proportion of citing papers used the frameworks for the express purpose of guiding an evaluation of research capacity in health settings. On the strength of this review, it appears that frameworks – even when developed within and for particular geographic and socioeconomic contexts – are useful in other contexts when used flexibly, modified and further developed [63]. In 2018, Luckson et al. [54] added a communication domain to Cooke’s earlier framework [17]. In 2021, Cooke developed and published an adapted version of their earlier framework [17], which focuses on research capacity development for impact [6]. In 2025, McGuire et al. [63] expanded Cooke’s later framework [6] by introducing an equity domain. These adaptations are essential for developing the field of RCB and evaluation processes, ensuring programmes achieve the end goal of producing research that is useful and is used.

The vast majority of citing papers featured the frameworks in what Brown and colleagues [82] likened to a “cameo role”, where the citation was made in the introductory or discussion sections of the paper only, to contextualize the study, define key concepts or add credibility. Authors who used the frameworks to guide their evaluation of research capacity did so in a variety of ways, drawing on one or more of the framework elements in their evaluation plans, with few using all framework components. This is consistent with other reviews of the use of evaluation frameworks [83]. Indeed, Cooke [17] advocated for the flexible use of their framework in a way that best suits the evaluation context. Differences in the extent and nature of the use of the frameworks reflects the variation in the priorities and characteristics of the context in which the evaluation of research capacity take place. This review did not set out to measure the quality or effectiveness of evaluation, and therefore, we cannot determine whether more extensive or complete use of a framework and its components leads to higher-quality evaluation. Nonetheless, one of the implications of using evaluation frameworks inconsistently and incompletely is the inability to compare the outcomes across individual evaluation studies, which may hinder the advancement of RCB theory and practice [83]. To enhance transparency in reporting, authors must indicate which components of the frameworks were used to evaluate RCB and why.

Further influencing variability in the use of the frameworks is the absence of a universal definition of research capacity building [31, 62]. Defining RCB is complicated by the ambiguity of the individual component, that is, what *research* is (and is not) and whether it encapsulates or is distinct from quality improvement, evidence-based practice and evaluation [84]. Further, what capacity means and how it relates to capability [85]. Although it is beyond the scope of this review to define RCB, it was evident that the framework authors defined or described RCB in nuanced ways – that there is common recognition of both individual and organization capacity, and that each definition featured the longer-term aim of enabling the conduct of useful health research that informs practice. The conduct of useful research is a somewhat intangible end point, given that usefulness is context-dependent, and informed by factors such as resourcing, research culture, and local and contemporaneous priorities, among other factors [2]. This reinforces the importance of aligning the application of the evaluation framework with the relevant organization’s strategy and interest-holder needs [49, 65, 67]. Engaging with interest-holders early to identify what is important and possible to measure is a key step in any evaluation [9, 13]. Enacting this engagement effectively, however, can be difficult in the context of RCB, where programmes tend to be developed, implemented and evaluated across multiple teams, organizations and jurisdictions, each with potentially unique needs and expectations that are shifting frequently in response to social and political influences [16, 86]. An alternative form of engagement with interest-holders is via published organizational strategies and other guidance documents, which may help those concerned with RCB to understand their priorities and synthesise these across multiple organizations. For example, Schmidt et al. [87] conducted a content analysis of a health organization’s strategic and operational documents. They examined the extent to which research activity was represented, the types of staff expected to engage in research and any other indications of research activity in the documents. This approach could be adapted and expanded to inform the development of an RCB evaluation plan.

The frameworks included in this review were developed through the collection and synthesis of retrospective and/or prospective data. On exploring the methods of development of other prominent frameworks, there was some variation observed. The authors of the RE-AIM framework for evaluating health promotion interventions [88] did not clearly articulate the approach used to develop the framework in their foundational paper. Conversely, the authors of the Theoretical Domains Framework [89] described the six-phase development process in detail, beginning with expert reviews of relevant theories and constructs, followed by validation through consensus with three expert groups. The Consolidated Framework for Implementation Research [90] was developed by an expert authorship team who identified and synthesised existing relevant theories. These methods of framework development are reflected to some extent in the articles included in the current review; however, in the absence of definitive best-practice guidelines for framework development, it is unclear whether any one method of, or approach to, framework development is superior, and therefore produces a higher-quality framework.

Many citing authors described the strengths of the frameworks in guiding a rigorous, comprehensive and nuanced evaluation that considered outcomes beyond traditional and so-called easy to measure research outputs. Further, many authors highlighted the role of frameworks in informing the development and implementation of RCB programmes and initiatives, and identifying barriers and enablers to research and RCB activities. Given that the frameworks were developed using evidence drawn from expert knowledge and experience, real-world case examples and research evidence, it is reasonable to suggest that evidence-based evaluation frameworks lend a greater level of rigour to the development and implementation of an evaluation plan. In turn, this will enable iterative programme re-development to ensure that the longer-term aims of RCB are achieved. Nonetheless, clear and compelling evidence that the use of an evaluation framework leads to better RCB outcomes has not been established through this review. Limitations of the frameworks were identified by both the authors themselves and citing authors. The former tended to focus on the methods of framework development, whereas the latter focused on real-world application. Although there have been adaptations to the frameworks over time, there is scope for further modernization and expansion of the frameworks by contributing additional domains through ongoing critical evaluation practice.

### Implications

We suggest those concerned with evaluating RCB in health settings should consider the following points:

1. Carefully consider and report the rationale for selecting a framework and detail the application of the framework, with reference to timing, elements of the framework used and not used, and any limitations or strengths identified to enhance evaluation transparency.
2. Engage with relevant interest-holders to identify which outcomes are important to work towards and measure; this may be through purposive synchronous engagement or by consulting published guidance and strategy documents that outline RCB priorities.
3. Critically engage with, modernize and expand on frameworks with a view to advance RCB evaluation theory and practice.

### Strengths and limitations of our review

This methodological review was undertaken using a systematic scoping approach to database and citation searching. Through these two methods of searching and review, we identified not only the frameworks but also explored how they have been applied in research, providing a comprehensive and nuanced understanding of the role and application of peer-reviewed, published frameworks to evaluate research capacity in health settings. Our team comprises numerous researchers with experience in developing, delivering and evaluating research capacity building programmes, and in health professions education research, contributing expert insights to the review. Nonetheless, this review has several limitations. The forward citation reviewing was conducted by a single author. This may have introduced some subjectivity in the identification and classification of how included frameworks were used. However, this component involved structured tool using predefined criteria and did not affect the primary study selection, which was conducted independently by two authors. This review is also limited by the concentration on peer-reviewed literature, excluding grey literature such as evaluation reports. This means published frameworks were excluded if they were not peer-reviewed, and non-peer-reviewed evaluation studies applying the frameworks were also excluded from the review. Furthermore, as this review focused on studies that cited the published frameworks in the Methods and Results sections, not all citing studies were included. In summary, this review does not include all published evaluations of research capacity, as it focused on peer-reviewed evaluation studies that used the identified peer-reviewed frameworks. The exclusion of non-peer-reviewed frameworks and citing evaluation studies limited our analysis of the nature of the use of all RCB evaluation frameworks in practice.

## Conclusions

There are currently nine peer-reviewed, published frameworks for evaluating research capacity. There are both commonalities and differences in the development and components of these frameworks. Citing authors used the frameworks to varying degrees and for different purposes, using different methods of data collection to suit their context. Framework authors and citing authors alike have identified limitations and strengths of the frameworks, with several examples of authors adapting and extending the frameworks using evidence generated in their own evaluation or in framework development research. This adaptation and modernization work reflects the changing health research and capacity building landscape and priorities and is essential to the advancement of RCB evaluation theory and practice. Cooke’s earlier framework remains the most frequently cited in the peer-reviewed literature, which may mean that important adaptations and additions to frameworks are not being applied in RCB evaluation practice. Researchers using a published framework to inform their evaluation of RCB should engage with their interest-holders in the development of their evaluation plan, consider and report their rationale for selecting their chosen framework, and critically engage with the framework to expand it, thereby advancing RCB evaluation theory and practice.

## Supplementary Information

Supplementary Material 1.

Supplementary Material 2

Supplementary Material 3.

Supplementary Material 4.

Supplementary Material 5.

## Data Availability

The authors confi rm that all data generated or analysed during this study are included in this published article from: 10.1186/s12961-026-01511-3.

## Abbreviations

RCB: Research capacity building
KATH: Komfo Anokye Teaching Hospital
RCC: Research Capacity and Culture (Tool)
