# Supplementary material for: The development and utility of frameworks designed to evaluate research capacity building initiatives in healthcare settings: a methodological review
Source: Health Res Policy Syst. 2026 Jul 16;24:60. doi: 10.1186/s12961-026-01511-3 (PMC13374067; doi:10.1186/s12961-026-01511-3)
Supplement: Supplementary file 2 — Supplementary Material 2 [file 12961_2026_1511_MOESM2_ESM.docx]

# Additional File 2 - Scoping Review Protocol

The review questions, objectives and inclusion/exclusion criteria were developed by the research team. These formed a protocol for undertaking a methodological review, drawing on some of the principles of systematic and scoping reviews [1, 2], using the Joanna Briggs Institute’s Methodology for Scoping Reviews [1].

## Review aim

To review the evidence on the development, components, and utilisation of frameworks designed to evaluate research capacity in health settings.

## Review purpose

To identify the processes utilised in the development of frameworks for evaluating RCB programs and initiatives implemented in healthcare settings; the key components of these frameworks; the evidence and theories underpinning, and utility of the frameworks as evidenced by forward citation and use by other researchers. This review will highlight the strengths and limitations of the published frameworks designed to guide the evaluation of research capacity building programs in health settings, and the strengths and limitations in their use in evaluation practice. This review will inform recommendations for the further development and use of RCB evaluation frameworks in practice.

## Scoping review objectives

To review the evidence on the:

- Development of evaluation frameworks for research capacity building programs and initiatives (e.g., education programs, mentoring, research funding initiatives) implemented in healthcare settings; including the methods used to develop the framework, the stakeholders involved and methods of stakeholder involvement
- Components of included evaluation frameworks
- Theories or evidence underpinning research capacity building evaluation frameworks
- Use of evaluation frameworks for research capacity building programs and initiatives implemented in healthcare settings.

## Review phases

This methodological review will be undertaken over two phases:

1. Systematic search of the peer-reviewed published literature to identify published frameworks designed to evaluate research capacity in health settings
2. A forward citation search of the frameworks identified as meeting the inclusion criteria for the first phase of this review, to identify the number of citing studies and investigate the use of the frameworks by citing authors

#### Table 1 Inclusion and exclusion criteria for Phase 1 of the review

| **Criteria** | **Inclusion** | **Exclusion** |
| --- | --- | --- |
| Types of **studies** | All types of studies that describe the development of new or modified evaluation frameworks, models, or strategies to guide the evaluation of outcomes or impacts of research capacity building programs, strategies, or initiatives implemented in health settings*  Research and quality improvement projects | Individual program evaluations that do not introduce or describe the development of a new evaluation framework, construct, or organising theory or structure; generic evaluation frameworks  PhD theses  Systematic reviews |
| Types of **data** | All forms of data used or described as part of the development of a framework to evaluate research capacity building programs delivered in healthcare settings (qualitative, quantitative, mixed, newly generated/primary data and secondary data); objective (e.g., metrics) and subjective (e.g., views/experiences) data | Papers presenting evaluation data with no reference to the development of the evaluation framework |
| Types of **methods** | Qualitative methods (interviews, focus groups, open text surveys, narrative or integrative literature reviews)  Quantitative methods (surveys, scales, meta-syntheses)  Consensus methods (Delphi studies, round table discussions, workshops)  Mixed methods approaches to developing frameworks | None |
| Types of **outcome** measures | Papers that describe any outcome measures as part of a research capacity building program evaluation framework | None |

*Health settings are defined as settings in which healthcare professionals work and deliver care and provide services to consumers/patients (e.g., hospitals, community health services, family practice, and clinics or surgeries)

## Databases to be searched

For Phase 1 of the review, the following research databases will be search for peer-reviewed literature: Ovid MEDLINE, CINAHL, PsycInfo, ERIC, Embase, Scopus, and Web of Science.

For Phase 2 of the review, citation searches of the included peer-reviewed frameworks will be conducted via Google Scholar.

## Data extraction

Data to be extracted from articles identified in both phases of the review will be input into two tables, and categorised under the following headings:

1. Citation; Name of framework; Year developed/published; Country and context of development/application; Definition of research capacity building; Mechanism of framework development (consensus, literature review, research, co-design, etc.); Underpinning evaluation or education/capacity (or other) building theory/ies; Strengths and limitations of evaluation framework (as reported by authors)
2. Citation; Name of framework; Overarching structural components of framework; Sub-structural components of framework; Outcome measures proposed/suggested in framework; Citations (n); Citing studies that meet Phase 2 inclusion criteria (n)
3. Citation; Name of citing author; Program name or research capacity evaluation context; Use or purpose of framework in the study; Study design and methods of data collection and analysis; Key findings/learnings reported; Strengths and limitations of evaluation framework (as reported by citing authors)

#### Table 2. Search strategy: Ovid MEDLINE

| **Master search for OVID Medline – 01/05/2025** | | |
| --- | --- | --- |
| **#** | **Search term** | **Results** |
| 1 | (capacit* adj2 (build* or strength or develop*)) | 22119 |
| 2 | Capacity Building/ | 38297 |
| 3 | 1 or 2 | 22119 |
| 4 | (framework* or tool* or indicator*).ab,ti. | 1950119 |
| 5 | (evaluat* or measur*).ab,ti. | 8299982 |
| 6 | Program Evaluation/ | 68982 |
| 7 | Evaluation Study/ | 265707 |
| 8 | 5 or 6 or 7 | 8435177 |
| 9 | research.ab,ti. | 2365373 |
| 10 | Health.ab,ti. | 2829152 |
| 11 | 9 and 10 | 536038 |
| 12 | Research Personnel/ or Research/ or Biomedical Research/ or Health Services Research/ | 336982 |
| 13 | 11 or 12 | 839434 |
| 14 | 3 and 4 and 8 and 13 | **870** |

## References

1. Colonna R, Knott M, Kim S, Bagajati R. Qualitative research on cannabis use among youth: a methodological review. Journal of drug issues. 2023;53(4):647-69.

2. Mbuagbaw L, Lawson DO, Puljak L, Allison DB, Thabane L. A tutorial on methodological studies: the what, when, how and why. BMC Medical Research Methodology. 2020;20(1):226.
