# Supplementary material for: The development and utility of frameworks designed to evaluate research capacity building initiatives in healthcare settings: a methodological review
Source: Health Res Policy Syst. 2026 Jul 16;24:60. doi: 10.1186/s12961-026-01511-3 (PMC13374067; doi:10.1186/s12961-026-01511-3)
Supplement: Supplementary file 3 — Supplementary Material 3. [file 12961_2026_1511_MOESM3_ESM.docx]

# Additional file 3 Search Strategy

**Aim:** To review the evidence on the development, components, and utilisation of frameworks designed to evaluate research capacity in health settings.

**Date:** 01/05/2025

| **Master search for OVID Medline** | | |
| --- | --- | --- |
| **#** | **Search term** | **Results** |
| 1 | (capacit* adj2 (build* or strength or develop*)) | 22119 |
| 2 | Capacity Building/ | 38297 |
| 3 | 1 or 2 | 22119 |
| 4 | (framework* or tool* or indicator*).ab,ti. | 1950119 |
| 5 | (evaluat* or measur*).ab,ti. | 8299982 |
| 6 | Program Evaluation/ | 68982 |
| 7 | Evaluation Study/ | 265707 |
| 8 | 5 or 6 or 7 | 8435177 |
| 9 | research.ab,ti. | 2365373 |
| 10 | Health.ab,ti. | 2829152 |
| 11 | 9 and 10 | 536038 |
| 12 | Research Personnel/ or Research/ or Biomedical Research/ or Health Services Research/ | 336982 |
| 13 | 11 or 12 | 839434 |
| 14 | 3 and 4 and 8 and 13 | **870** |
