# Supplementary material for: The development and utility of frameworks designed to evaluate research capacity building initiatives in healthcare settings: a methodological review
Source: Health Res Policy Syst. 2026 Jul 16;24:60. doi: 10.1186/s12961-026-01511-3 (PMC13374067; doi:10.1186/s12961-026-01511-3)
Supplement: Supplementary file 4 — Supplementary Material 4. [file 12961_2026_1511_MOESM4_ESM.docx]

# Additional File 4 – Excluded Framework Articles

| **Reference** | **Reason for exclusion** |
| --- | --- |
| Ali J, Kass NE, Sewankambo NK, White TD, Hyder AA. Evaluating international research ethics capacity development: an empirical approach. Journal of Empirical Research on Human Research Ethics. 2014 Apr;9(2):41-51. | Not health setting |
| El Achi N, Papamichail A, Rizk A, Lindsay H, Menassa M, Abdul-Khalek RA, Ekzayez A, Dewachi O, Patel P. A conceptual framework for capacity strengthening of health research in conflict: the case of the Middle East and North Africa region. Globalization and health. 2019 Nov 28;15(1):81. | Not health setting |
| Kasprowicz VO, Jeffery C, Mbuvi D, Bukirwa V, Ouattara K, Kirimi F, Heitz-Tokpa K, Gorrethy M, Chopera D, Nakanjako D, Bonfoh B. How to improve research capacity strengthening efforts: learning from the monitoring and evaluation of four research consortia in Africa. Health research policy and systems. 2023 Oct 25;21(1):109. | Not health setting |
| Chi BH, Belizan JM, Blas MM, Chuang A, Wilson MD, Chibwesha CJ, Farquhar C, Cohen CR, Raj T. Evaluating academic mentorship programs in low-and middle-income country institutions: proposed framework and metrics. The American journal of tropical medicine and hygiene. 2018 Nov 14;100(1 Suppl):36. | Not health setting |
| Tagoe N, Pulford J, Kinyanjui S, Molyneux S. A framework for managing health research capacity strengthening consortia: addressing tensions and enhancing capacity outcomes. BMJ Global Health. 2022 Oct 1;7(10):e009472. | Not health setting |
| Pulford J, Crossman S, Abomo P, Quach JA, Begg S, Ding Y, El Hajj T, Bates I. Guidance and conceptual tools to inform the design, selection and evaluation of research capacity strengthening interventions. BMJ Global health. 2021 Mar 1;6(3):e005153. | Not health setting |
| Scarlett J, Forsberg BC, Biermann O, Kuchenmüller T, El-Khatib Z. Indicators to evaluate organisational knowledge brokers: a scoping review. Health Research Policy and Systems. 2020 Aug 24;18(1):93. | Not health setting |
| Bates I, Boyd A, Smith H, Cole DC. A practical and systematic approach to organisational capacity strengthening for research in the health sector in Africa. Health research policy and systems. 2014 Mar 3;12(1):11. | Not an evaluation framework |
| Matus J, Walker A, Mickan S. Research capacity building frameworks for allied health professionals–a systematic review. BMC health services research. 2018 Sep 15;18(1):716. | Not an evaluation framework |
| Whiteside M, Smith R, Gazarek J, Bridge F, Shields N. A framework for enabling evidence-based practice in allied health. Australian Social Work. 2016 Oct 1;69(4):417-27. | Not an evaluation framework |
| Brazil K. A framework for developing evaluation capacity in health care settings. Leadership in Health Services. 1999 Mar 1;12(1):6-11. | Not an evaluation framework |
| Holden L, Pager S, Golenko X, Ware RS. Validation of the research capacity and culture (RCC) tool: measuring RCC at individual, team and organisation levels. Australian journal of primary health. 2012 Mar 21;18(1):62-7. | Not an evaluation framework |
| Huber J, Bauer D, Hoelscher M, Kapungu J, Kroidl A, Lennemann T, Maganga L, Opitz O, Salehe O, Sigauke A, Fischer MR. Evaluation of health research capacity strengthening trainings on individual level: validation of a questionnaire. Journal of evaluation in clinical practice. 2014 Aug;20(4):390-5. | Not an evaluation framework |
| Cole DC, Kakuma R, Fonn S, Izugbara C, Thorogood M, Bates I. Evaluations of health research capacity development: a review of the evidence. Am J Trop Med Hyg. 2012;87(5 Suppl 1):226-305. | Review of evaluation studies or frameworks |
| Huber J, Nepal S, Bauer D, Wessels I, Fischer MR, Kiessling C. Tools and instruments for needs assessment, monitoring and evaluation of health research capacity development activities at the individual and organizational level: a systematic review. Health research policy and systems. 2015 Dec 21;13(1):80. | Review of evaluation studies or frameworks |
| Mugabo L, Rouleau D, Odhiambo J, Nisingizwe MP, Amoroso C, Barebwanuwe P, Warugaba C, Habumugisha L, Hedt-Gauthier BL. Approaches and impact of non-academic research capacity strengthening training models in sub-Saharan Africa: a systematic review. Health research policy and systems. 2015 Jun 9;13(1):30. | Review of evaluation studies or frameworks |
| Slade SC, Philip K, Morris ME. Frameworks for embedding a research culture in allied health practice: a rapid review. Health research policy and systems. 2018 Mar 21;16(1):29. | Review of evaluation studies or frameworks |
| Boyd A, Cole DC, Cho DB, Aslanyan G, Bates I. Frameworks for evaluating health research capacity strengthening: a qualitative study. Health research policy and systems. 2013 Dec 14;11(1):46. | Review of evaluation studies or frameworks |
| Bates I. Designing and measuring the progress and impact of health research capacity strengthening initiatives. In BMC proceedings 2015 Dec 18 (Vol. 9, No. Suppl 10, p. S9). London: BioMed Central. | Review of evaluation studies or frameworks |
| Levine R, Russ-Eft D, Burling A, Stephens J, Downey J. Evaluating health services research capacity building programs: implications for health services and human resource development. Evaluation and program planning. 2013 Apr 1;37:1-1. | Individual program evaluation |
| Tajuria G, Dobel-Ober D, Bradley E, Charnley C, Lambley-Burke R, Mallen C, Honeyford K, Kingstone T. Evaluating the impact of the supporting the advancement of research skills (STARS) programme on research knowledge, engagement and capacity-building in a health and social care organisation in England. BMC Medical Education. 2024 Feb 8;24(1):126. | Individual program evaluation |
| Maige, J. S.; Mosha, A. S.; Mero, V. A.; Kiware, S. S. Research capacity monitoring and evaluation system: A robust dynamic web-based application for measuring the impact of research capacity strengthening initiatives. American Journal of Tropical Medicine and Hygiene. 2023 (Vol. 108, No. 4, p.220) | Insufficient details |
| Ansong D, Purnell S, Bedu-Addo G, Osei-Akoto AY, Karikari P, Agbenyega T, Bates I. Strengthening research capacity within a ghanaian teaching hospital: Ten year prospective study. The American Society of Tropical Medicine and Hygiene Annual Meeting. 2012 (Vol 87, No. 5, Suppl, p. 242) | Insufficient details |
