# Supplementary material for: The development and utility of frameworks designed to evaluate research capacity building initiatives in healthcare settings: a methodological review
Source: Health Res Policy Syst. 2026 Jul 16;24:60. doi: 10.1186/s12961-026-01511-3 (PMC13374067; doi:10.1186/s12961-026-01511-3)
Supplement: Supplementary file 5 — Supplementary Material 5. [file 12961_2026_1511_MOESM5_ESM.docx]

# Additional file 5 - List of All Example Indicators and Outcomes

**Cooke, 2005 [1]**

**Building skills and confidence***Individual:* Skills developed; Progressive skill development; Confidence building (e.g., via sharing new skills with others, applying existing skills in new situations, new research collaborations); Research undertaken

*Teams:* Skills developed; Skill mix of team; Skill/knowledge transfer tracked/audited; Progressive skill development; Confidence building (e.g., via sharing new skills with others, applying existing skills in new situations, new research collaborations)
*Organisational:* Training research needs assessment completed; Availability and use of research training funds; Evidence of outreach capacity building initiatives; Levels of skills and skill mix across the workforce; Connections between novice and experienced researchers; Research undertaken, funding approved
*Supra organisational (networks and support units)***:** Delivery of flexible learning packages; Delivery of research training tailored to the needs of professional groups; Examples of knowledge/information transfer (e.g., via workshops and discussions forums); Evidence of outreach capacity building initiatives and uptake of these; Responses to needs based work; Secondment opportunities available and taken up
**Close to practice**
*Individuals and Teams:* Clinical expertise driving research projects; Examples of critical thinking used in practice; Patient centred outcome measures in projects, and impact of project on patients' quality of life; Use of action orientated research methods; Use of cost effectiveness methods and approaches; Evidence of service user involvement in research
*Organisational:* Organisational knowledge gaps inform research questions and project; Research culture evaluation (i.e., research is valued, accepted, encouraged, etc.): Overt managerial support for/involvement in research projects; Service user involvement in research
*Supra organisational (networks and support units):* Research questions/projects informed by practice needs and priorities; Co-ordination of research between health organisations and universities; Development and use of outcomes measures useful for research and practice; Development and use of cost effectiveness research methods; Action research orientated approaches undertaken; Service user panels established
**Linkages, collaborations and partnerships**
*Individual:* Participation in partnerships to gain and share knowledge; Increased number of research partnerships; Interprofessional working
*Teams:* Who the team has worked with (academic and practice); Network development; Inter-professional and other collaborative working
*Organisational:* Collaborations with universities and research development units; Joint positions with universities; Collaborative research with other health services; Contributions/memberships with networks; Work with funding bodies
*Supra organisational (networks and support units):* Established join posts; Research collaboration with practitioners/ teams/ networks/ health organisations; Partnerships and collaborations across networks; International links
**Appropriate dissemination and impact***Individuals and Teams:* Journal articles published; Conference presentations; Applied dissemination of findings; Influence of research on local strategy/planning
*Organisational:* Access to findings of research undertaken locally; Seminars/showcases to share local research activity and findings; Application of research findings to practice locally; Funding to support research dissemination; Applications for intellectual property for research completed locally
*Supra organisational (networks and support units):* Practitioner-led health services research published; Presentations at practice- focussed conferences; Applied dissemination of research; Innovative dissemination of research; Applications for intellectual property for research completed locally
**Continuity and sustainability**
*Individual:* Access to funding for continued application of skills (e.g., grants and fellowships); Continued contacts with collaborators/partners; Continued support and supervision arrangements
*Teams:* Recognition and matching of skills; Access to funding for continued application of skills (e.g., grants and fellowships)
*Organisational:* Secondment opportunities available and used; Access and uptake of local responsive funding; Recognition and matching of skills; Examples of continued collaboration
*Supra organisational (networks and support units)*: Continued collaborations; Supported research career pathways; Fellowships supported
**Infrastructure**
*Individual:* Project management parameters within projects (e.g., objectives and timeframes); Mentorship and supervision structures; Job descriptions and annual appraisals include research
*Teams:* Project management parameters within projects (e.g., objectives and timeframes); Mentorship and supervision structures; Protected research time utilisation
*Organisational:* Research and development information dissemination strategies; Protected research time available and used; Back fill time available and used; Research part of annual appraisal for some jobs; Support available for governance and ethics processes
*Supra organisational (networks and support units):* The nature of collaborations (co-authorship, order of authorship); Information exchange events (attendance)

**Bates, 2006 [2]**

**Awareness:** Funding commitments from national and international partners for RCB; Partners’ roles and responsibilities defined
**Implementation:** Improved institutional research support, research group, and local research funds; Researchers awarded diploma (n)
**Expansion:** Broad research scope within and beyond the hospital; Quality assurance education processes in place; Research findings incorporated into audit cycles; Long-term funding secured; hospital-led research papers published and grants obtained
**Consolidation:** Health research included in undergraduate/postgraduate curricula; Departmental budgets allocated for research activities and outputs

**Sarre, 2009 [3]**

**Infrastructure:** Research support roles (n); Organisational investment to promote research; Accessible guidance for researchers

**Linkages and partnerships:** Links with research development and support units; Membership/partnership with research alliances/consortia; Conjoint posts with academic institutions (n)

**Skills development:** Research secondment opportunities available and taken up (n); Research training needs assessments and delivery; Fellowships, higher degree research programs undertaken by health staff (n)

**Dissemination:** Database of organisation's projects and findings; Organisational events for local research dissemination; Annual reports featuring research findings

**Research activity:** Number/level of research activity/ies in the organisation; Chief/principal investigators employed by the organisation (n); National collaborative projects organisation involved in (n)

**Close to practice:** Projects with user involvement (n); Projects with health staff collaboration (n); Evidence of use of local research to inform organisation's practice

**Continuity and sustainability:** Strategy includes recurrent investment in research development; Evidence organisation supports application for external funding

**Leadership:** Presence of a director of research and development; Frequency of reporting research activity and RCB at Board level

**Research culture:** Celebration of research achievements; Marketing of research for new staff recruitment; Manager attendance at research events

**Bates, 2011 [4]**

**Awareness:** List of capacity gaps to be filled; List of stakeholders critical for implementing project outputs; Evidence of stakeholder engagement for RCB activities
**Experiential:** Written plan and timeframe for addressing gaps; Strategy for using research outputs to rectify gaps between evidence and policy/practice; Outcomes from testing of pilot projects/models for RCB
**Expansion:** Skill and workforce expansion; Reduced reliance on northern partners; Review process for updating/developing policies; Evidence of strengthened of systems (e.g. new committees or reporting structures); Diversification of funding sources; Publications and/or presentations at national/international meetings
**Consolidation:** Evidence long-term funding secured; Project management and key decisions led by southern partners

**Cole, 2014 [5]**

**Individual level indicators of research skills training**

*Activities:* PhDs, Master of Science courses, fellowships, training for research support staff

*Outputs:* Recipients’ feedback about career prospects; Quality of training; Balance between training in research methods, research process and advocacy, promotion, negotiation, and resource mobilisation
*Outcomes:* Development of research skills (e.g., research problem identification, critical review of a research paper, research proposal, and report writing); Quantitative and qualitative evidence of award’s effectiveness (via surveys); Evidence awardees continued active independent research in LMIC; Reasons trainees left LMICs; Sustained research collaborations; HIC researchers’ improved understanding of international issues and increased desire to collaborate with researchers in LMICs; New research funding obtained
**Individual level indicators of mentoring**

*Activities:* Individual support and supervision for researcher development

*Outputs:* Trainees with a mentor (n); Reasons for lack of career development
*Outcomes:* Grantees working as senior researchers and their location (n); Percentage time spent engaged in research
**Individual level indicators of course and curricula development**

*Activities:* Short courses in research methods/skills developed in response to needs assessment and embedded in university

*Outputs:* Partnerships used for course design, student supervision, mentoring, and bilateral recognition of credits; Research courses run by university consortia-promoted relationships; Database of courses, attendance register

*Outcomes:* Secondary benefits to students (e.g., travel and education opportunities), students become knowledge “diffusers”
**Individual level indicators of scientific conference and workshop**

*Activities:* Health economics conference, forums, networking and policy makers

*Outputs:* Meetings/workshops attended pre- and post-funding

*Outcomes:* Awardees research published in conference proceedings; Speaking invitations; Honours, awards; Expanded networks; Membership/leadership roles in professional societies, advisory groups or scientific journals
**Institutional level indicators of human resources strengthening**

*Activities:* Staff training, recruitment, salaries; Strengthening relationships between staff and students; Promoting interdisciplinarity, diversity and specialisation

*Outputs:* Potential supervisors (n); Capacity to mentor junior researchers and take leadership roles; Institutional destinations/returned researchers and graduates

*Outcomes:* Recruitment/retention researchers, supervisors, core staff; Research career pathways; Research managers involved in collaborations/networks

**Institutional level indicators of activities for strengthening research infrastructure and management** *Activities:* Infrastructure (lab facilities, IT, etc.); Ethical review boards set up; stakeholder engagement; Improved governance, financial reporting; Institutional evaluation capacity; Analyse gender equity

*Outputs:* Cross-cutting projects established, sharing equipment, staff, and systems/facilitates, integrated research activities; Standard operating procedures, quality assurance mechanisms;
Research support centre, scientific steering committee, institutional governance structure, and organisational chart; Strategic planning, management, new policies, resource allocations; Evidence of a transferable partly self-sustaining model for Research Support Centre
*Outcomes:* Access to resources (e.g., staff, libraries, journals, equipment); Research staff satisfaction with institution’s research services; Improved management, administrative and technical capacities; International accreditation achieved
**Institutional level indicators of scientific collaboration**

*Activities:* Promote North-South and South-South collaborations and regional partnerships

*Outputs:* Formal data sharing agreements; Joint site inspections and meetings; Joint PhD students, projects, and technologies between collaborators
*Outcomes****:***  Trust in and commitment to collaborations that continue after award concludes; Benefits to northern institutions

**National-international level indicators of engagement and communication activities for research uptake**

*Activities:* Engage with private and non-health organisations, NGOs, health programs, research institutions, health ministries, regulatory authorities, via journals, press, magazines, conferences, workshops, networks, websites, and other media

*Outputs:* Skills development program from public-private-academic partnerships; Plan for acquiring and using research information, and for sharing knowledge; Media articles; Communication/ knowledge management strategy; Trends in website access
*Outcomes:* Enhanced health research capacity strengthening (RCS) effort or knowledge of neglected topic diseases; Knowledge of focus of health RCS efforts; Partnerships for research dialogue (e.g., with policymakers, research users, decision makers national authorities, etc.) at local, regional, and international levels
**National-international level indicators of activities to develop national health research systems or scientific councils**

*Activities:* Promote financial sustainability in regional research activities

*Outputs:* National research system map
*Outcomes:* Commitment and active engagement by national health research institutions and health ministries to review progress and determine research priorities; Knowledge of contributions of agencies to national health research system and creating demand for research; External accessible and flexible funds **National-international level indicators of networking activities for researchers and/or research users**

*Activities:* Facilitate collaborations and networks (e.g., though multidisciplinary workshops, curricula, meetings, etc.)

*Outputs:* New research partnership to strengthen links between universities and policy-making; Project staff contributed to evaluations of health centres and systems; North–South and South–South networking activities; Active committees with institutional representation in each member country; Commitment and communication with the Northern and among Southern partners
*Outcomes:* Policy, practice, and knowledge impacts at different levels (i.e., international, regional, national, district level) and on health and non-health sectors; Estimated impact on disease management and prevention; Regional research activities

**Murphy, 2015 [6]**

**Research-supportive organisational policies and procedures:** New structures, policies, and procedures to support research activity
**Staff knowledge, skills, and confidence to access and use research:** Attendance at and satisfaction with research interest group meetings; Increased mean knowledge scores and self-ratings for research training events; Increased mean research knowledge ratings in all health service departments
**Involvement of health service staff in research activity:** New projects initiated; New collaborations; Increased number of staff involved in research including presentations and publications **Research communication research within health service:** Research update section in every health service newsletter; 50% more survey respondents aware of RCB with example of activity over 2-year period; Monthly health service research reports submitted to relevant committees; Research as a standing agenda item at relevant committee meetings
**Research informs organisational governance documents:** One or more governance document informed by research findings **Health service staff work satisfaction:** Increased mean work satisfaction in all health service departments **Use of research in health practice:** Evidence-based actions/decisions as an outcome of each research project

**Pulford, 2020 [7]**

**Individual level***Bibliometrics:* Number of peer-reviewed publications; Conference presentations; Citations**;** Publications with impact factor indexed in Web of Science
*Collaborative activities*: Evidence of engagement in or with networks; Development and sustainment of research collaborations; Attitudes/behaviour conducive to effective partnerships and working toward RCB goals
*Knowledge translation:* Dissemination of findings; Evidence of influence on strategy/planning
*Re5-yearion:* Editor conference proceedings; Number/type of awards; Invitations to speak/present at conferences/meetings (n)
*Research funding:* New research funds received
*Research Management Systems:* Percentage time spent on research activity
*Skills/knowledge:* Using new evaluation method; Evidence progressive skill development; Learnings shared with others in the organisation
*Other:* Scientific merit of research proposal; Number of grants produced; Engagement with end-users in research planning/design; Evidence of return/retention to research teams in LMICs; Research leadership roles in networks and communities of practice
**Institutional level***Bibliometrics:* Number collaborative publications; Number research reports; High quality literature reviews
*Collaboration activities:* Collaborative activities with other organisations (n); Research networks within and across institutions; Sustained collaborations with inherent trust and commitment
*Knowledge translation:* Knowledge exchange events (n); Exemplars of application of research to strategy/policy/practice
*Recognition:* Improved reputations
*Research funding:* Funding secured for research and RCB; Allocated budget to priority research activity; Funding spent in alignment with workplans
*Research Management Systems:* Evidence data systems used for reporting within the organisaton; Investment in research dissemination; Evidence of connections between novice and more experienced researchers; Access to technology to support research; Financial sustainability; Development of institution according to its visions/mission
*Skills/knowledge:* New skills applied to financial management of research; Increased capacity to conduct high quality evaluation; Advising/preparing higher degree by research students about research design/protocols
*Other:* Quality research outputs; Evidence of secondment opportunities available/taken up; Range and scale of research activities; Workforce skill mix; Evidence of processes to support end-user engagement in research
**Systemic level***Bibliometrics:* Proportion of publications with a first author from the institution
*Collaboration activities:* Changes to the way organisations collaborate and share information/knowledge; Research partnerships communication at local, regional and international levels; Partnerships perceived as useful
*Knowledge translation:* Media interest in research; Research influences policy decisions
*Recognition:* Increased respect between organisations and more reciprocal learning activities
*Research funding:* Government funding for research; Access and use of local funds
*Research Management Systems:* Ownership of research and research management systems locally; Coordinated regional research activities; Research ethics and governance systems in place; Salary equity for researchers
*Skills/knowledge:* Diffusion of research knowledge/skills via student researchers
*Other***:** Addressing regional problems through research; Evidence of retention of experienced researchers; Coordinated research efforts across the region; equitable access to knowledge and experience; Satisfaction among staff; importance of multidisciplinary research over 5 year period

**Sabey, 2023 [8]**

**Individual level, Immediately post-training:**•Increased knowledge/understanding
•Increased confidence in topic/activity
•Increased capability to apply learnings
**Individual level, Immediate/Medium term:
•**Intentions to change practices/behaviours
**Individual level, Medium/Longer term:
•**Value of training/learning to career
**Group or organisation, Immediate/Medium term:
•**New networks/collaborations as a result of training
•Evidence of planned or actual sharing of knowledge/learnings within the organisation
**Group or organisation, Medium term:
•**Changes in work practices, systems, decision-making, policy following the training **Group or organisation, Medium/Longer term:
•**Increased research/evaluation activity in organisation as a result of training
•Improved and sustainable organisational research culture
**Health and care system level, Longer term
•**New cross-system collaboration since training
•Embedded research/evaluation training across system

Note: Cooke’s 2021 framework [9] did not include a list of outcomes or indicators.

### References

1. Cooke J. A framework to evaluate research capacity building in health care. BMC Family Practice. 2005;6.

2. Bates I, Akoto AYO, Ansong D, Karikari P, Bedu-Addo G, Critchley J, et al. Evaluating health research capacity building: An evidence-based tool. PLoS Medicine. 2006;3(8):1224-9.

3. Sarre G, Cooke J. Developing indicators for measuring research capacity development in primary care organizations: A consensus approach using a nominal group technique. Health & Social Care in the Community. 2009;17(3):244-53.

4. Bates I, Taegtmeyer M, Squire SB, Ansong D, Nhlema-Simwaka B, Baba A, et al. Indicators of sustainable capacity building for health research: analysis of four African case studies. Health Research Policy and Systems. 2011;9.

5. Cole DC, Boyd A, Aslanyan G, Bates I. Indicators for tracking programmes to strengthen health research capacity in lower- and middle-income countries: a qualitative synthesis. Health Research Policy & Systems. 2014;12:17.

6. Murphy K, Stockton D, Kolbe A, Hulme-Chambers A, Smythe G. Building Research Capacity in a Regional Australian Health Service: a management strategy analysis. Asia Pacific Journal of Health Management. 2015;10(1):14-22.

7. Pulford J, Price N, Quach JA, Bates I. Measuring the outcome and impact of research capacity strengthening initiatives: A review of indicators used or described in the published and grey literature. F1000Research. 2020;9:517.

8. Sabey A, Biddle M, Bray I. Developing a framework to guide the evaluation of training in research skills for health and care professionals. Education for Health. 2023;36(2):83-7.

9. Cooke J. Building Research Capacity for Impact in Applied Health Services Research Partnerships Comment on" Experience of Health Leadership in Partnering With University-Based Researchers in Canada–A Call to" Re-imagine" Research". International Journal of Health Policy and Management. 2021;10(2):93.
